# A Systematic Review and Critical Assessment of Breast Cancer Risk Prediction Tools Incorporating a Polygenic Risk Score for the General Population

**DOI:** 10.3390/cancers15225380

**Published:** 2023-11-12

**Authors:** Cynthia Mbuya-Bienge, Nora Pashayan, Cornelia D. Kazemali, Julie Lapointe, Jacques Simard, Hermann Nabi

**Affiliations:** 1 Department of Social and Preventive Medicine, Faculty of Medicine, Université Laval, Quebec City, QC G1V 0A6, Canada; cynthia.mbuya-bienge.1@ulaval.ca (C.M.-B.); cornelia.kazemali.1@ulaval.ca (C.D.K.); 2 Oncology Division, CHU de Québec-Université Laval Research Center, Quebec City, QC G1S 4L8, Canada; julie.lapointe@crchudequebec.ulaval.ca; 3 Department of Applied Health Research, University College London, London WC1E 6BT, UK; n.pashayan@ucl.ac.uk; 4 Endocrinology and Nephology Division, CHU de Québec-Université Laval Research Center, Quebec City, QC G1V 4G2, Canada; jacques.simard@crchudequebec.ulaval.ca; 5 Department of Molecular Medicine, Faculty of Medicine, Université Laval, Quebec City, QC G1V 0A6, Canada

**Keywords:** breast cancer, polygenic risk score (PRS), risk prediction tools, non-genetic risk factors, systematic review

## Abstract

**Simple Summary:**

Several risk prediction tools have been developed to better stratify women according to their risk of developing breast cancer (BC) and inform prevention and early detection strategies. Many recent versions of these tools now incorporate a polygenic risk score (PRS) that uses the aggregated effect of common genetic variants, also known as single nucleotide polymorphisms (SNP), as a reliable predictor to estimate BC risk. However, the characteristics of each tool in terms of PRS development, population, and risk factors included vary considerably, which may affect their predictive performance and limit their use in public health practices. Thus, this systematic review characterizes BC risk prediction tools incorporating a PRS and explores the factors that can influence their ability to predict a woman’s risk of developing BC during her lifetime.

**Abstract:**

Single nucleotide polymorphisms (SNPs) in the form of a polygenic risk score (PRS) have emerged as a promising factor that could improve the predictive performance of breast cancer (BC) risk prediction tools. This study aims to appraise and critically assess the current evidence on these tools. Studies were identified using Medline, EMBASE and the Cochrane Library up to November 2022 and were included if they described the development and/ or validation of a BC risk prediction model using a PRS for women of the general population and if they reported a measure of predictive performance. We identified 37 articles, of which 29 combined genetic and non-genetic risk factors using seven different risk prediction tools. Most models (55.0%) were developed on populations from European ancestry and performed better than those developed on populations from other ancestry groups. Regardless of the number of SNPs in each PRS, models combining a PRS with genetic and non-genetic risk factors generally had better discriminatory accuracy (AUC from 0.52 to 0.77) than those using a PRS alone (AUC from 0.48 to 0.68). The overall risk of bias was considered low in most studies. BC risk prediction tools combining a PRS with genetic and non-genetic risk factors provided better discriminative accuracy than either used alone. Further studies are needed to cross-compare their clinical utility and readiness for implementation in public health practices.

## 1. Introduction

Breast cancer (BC) remains a major public health issue worldwide and is the second leading cause of death among women annually [1]. There is compelling evidence that early detection by mammography screening improves prognosis and reduces mortality rates from BC even though risks of overdiagnosis, over-treatment and psychological impacts cannot be discounted [2,3].

Currently, most organized BC screening programs are offered to women based solely on their age, from age 40–50 to age 70–74, depending on the country [4,5]. Although the risk of developing BC increases with age, genetic, environmental, lifestyle, reproductive and hormonal factors have been found to be associated with the risk of developing the disease [6]. In this context, risk-stratified BC screening, in which individual risk assessment based on multiple risk factors is used to tailor screening recommendations (e.g., more screening for women at higher risk and less screening for those at lower risk), has been proposed as an alternative to the current age-based approach [7,8,9]. Developing and validating accurate BC risk prediction tools is therefore critical to achieving optimal risk-stratified BC screening strategies.

Genome-wide association studies (GWAS) have identified common, low-penetrance genetic variants associated with BC risk [10,11]. Although individually, these variants confer minimal risk of BC, their effect becomes significant when aggregated as a polygenic risk score [(PRS), also known as genetic risk score—GRS] [12]. This PRS can be used alone or incorporated into a risk prediction model to identify women at higher risk of developing BC [13,14]. At the population level, risk prediction tools could be used to stratify healthy women based on their risk level of developing cancer in a certain time period (commonly 5 or 10 years) in order to adapt preventive measures [15,16,17]. While risk prediction models aim to predict the probability of an event occurring in individuals based on a combination of factors, risk prediction tools are the means by which these models are implemented in clinical or public health practice [18]. Commonly used tools to predict BC risk include the Breast Cancer Risk Assessment Tool (BCRAT; also referred to as the Gail model) [19], the International Breast Cancer Intervention Study model (IBIS; the Tyrer–Cuzick model) [20], BRCAPRO risk assessment tool [21], and the Breast and Ovarian Analysis of Disease Incidence and Carrier Estimation Algorithm model (BOADICEA) [13,22]. Clinical-grade tests to measure PRS are now available, and several BC risk prediction tools, including IBIS and BOACIDEA, have been extended to include a PRS value [13,23,24,25].

For a risk prediction tool based on or incorporating a PRS to be clinically useful for prevention or early detection, it must provide good risk discrimination between individuals who will develop the disease and those who will not and account for the population risk of the disease. Even if a model is well-calibrated to predict different risk categories, its ability to stratify groups of individuals in the population with a sufficient difference in absolute risk to justify additional preventive interventions plays an important role in its clinical utility [23]. Nonetheless, considerable heterogeneity in PRS development methods brings uncertainty and potential bias to BC risk prediction tools incorporating a PRS [26].

To date, there is no critical assessment regarding the development, performance, and risk of bias of BC risk prediction tools that include a PRS. In addition, population characteristics for which these tools are best suited remain to be further investigated. Finally, we need to understand the current validation processes of these tools and the existence of comparative studies to envision how they could apply to clinical or public health practices. Thus, we conducted this systematic review to help fill this gap in the literature. Our specific aims are to (1) identify, characterize, and summarise the different prediction risk models incorporating a PRS to estimate the risk of developing BC in women in the general population; and (2) assess the risk of bias of individual studies reporting on their performance.

## 2. Methods

### 2.1. Protocol and Registration

A systematic review protocol was published in the International Prospective Register of Systematic Reviews PROSPERO (PROSPERO 2020 CRD42020198930 available at https://www.crd.york.ac.uk/prospero/display_record.php?RecordID=198930 (accessed on 6 November 2023).

### 2.2. Search Strategy

This systematic review followed PICOTS and PRISMA guidelines [27,28]. We proceeded to a first search of the Medline, EMBASE databases and the Cochrane Library up to June 2021 using the strategy presented in Supplemental Table S1. We then updated our search to retrieve relevant literature up to November 2022. Our search strategy, adapted for each database, consisted of a combination of keywords and controlled vocabulary for three concepts: “breast cancer”, “polygenic risk score or genetic risk score” and “cancer risk prediction tools”. We also manually screened bibliographic references of all included papers and other relevant systematic reviews or meta-analyses to retrieve additional studies.

### 2.3. Eligibility Criteria

We included studies reporting original research published in a peer-reviewed journal describing the development and/or validation of prediction models incorporating a PRS and using it to estimate the risk of developing BC for adult women in the general population. We defined the general population as a cohort representing women typically considered at average risk of developing BC. Therefore, we excluded studies including individuals with a history of BC or focusing on specific population groups (e.g., individuals with a known mutation in *BRCA1* or *BRCA2* genes, nurses’ study, hereditary BC risk in a familial setting, etc.). The prediction models included in this review could either use only genetic factors in the form of a PRS or a combination of genetic and non-genetic risk factors. To be included in our review, studies needed to meet the following criteria: (1) describe the development and/or validation of a prediction model; (2) use at least two SNPs in the form of a PRS or GRS; (3) predict the risk of developing BC for a specified period in an individual’s life (e.g., 5 or 10-year risk, lifetime or remaining lifetime risk); (4) report a measure of performance to assess the predictive capacity of the model (i.e., measure of discrimination (e.g., C-statistic, AUC), or calibration (e.g., Hosmer–Lemeshow statistic)). Articles published in French or English and with any study design were considered without restriction on the publication year.

### 2.4. Study Selection

Two independent reviewers (C. Mbuya-Bienge and C.D. Kazemali) screened the titles and abstracts. A first pilot selection of titles and abstracts was conducted from a random sample of 10% of the identified articles to verify the clarity and consistency of inclusion criteria. Since the kappa statistic was 0.9 for this pilot, indicating no significant problems, no changes were made to the selection process and criteria. The full text of all potentially relevant studies was also assessed independently by the two researchers. When a consensus to include or exclude a study could not be reached, a senior researcher (H. Nabi) made the final decision.

### 2.5. Data Extraction Process and Analysis

Data extraction was undertaken independently by the same two researchers (C. Mbuya-Bienge and C.D. Kazemali) using a grid based on the CHARMS checklist of relevant items to extract from individual studies of prediction tools [29]. Data were extracted into tables divided into four categories that influence the models’ validity and utility: (1) study characteristics, (2) outcomes and predictors, (3) model development and (4) model performance and validation. Study characteristics included information such as study design, source of data, study population size, study population characteristics (e.g., ethnicity) and type of study according to the TRIPOD guidelines [30] (e.g., development only (1a); development and validation using resampling (1b); random (2a) or non-random (2b) split sample development and validation; development and validation using a separate data (3); and validation only (4)). Outcomes and predictors were assessed based on the studies’ definition and method for measuring the outcomes and predictors, handling of predictors and selecting genetic and non-genetic predictors. For the model development phase, we considered the handling of missing data, the modelling method and the model presentation. Model performance and validation were assessed from the reported measure of performance, the classification measures if available and the method of internal or external validation if applicable.

The heterogeneity of model characteristics in terms of predictors and outcomes precluded the possibility of pooling data across studies. Therefore, a narrative synthesis was conducted. Key study characteristics, validation and accuracy of individual risk prediction models, as well as the methodological quality, are described in tables and summarised narratively. We presented the studies’ main measure of discrimination and its 95% confidence interval (CI), when provided, using a forest plot. The measures of discrimination, such as the area under the receiver operating characteristics curve (AUROC or AUC) or the concordance statistics (c-statistics), indicate how well patients can be classified into two groups (usually the cases with the disease and the controls without the disease). Possible values range between 0.0 and 1.0 with a value of 1.0 indicating that the model has a perfect classification accuracy and 0.5 indicating that the classification is not better than a random classification. On rare occasions, the value can be less than 0.5, indicating that the model has an inaccurate classification accuracy (i.e., it performs worse than chance) [31,32]. When the same study reported performance measures for multiple steps of the same model (e.g., model development and internal validation), only the best-performing model was included in our main analysis. When an article presented performance measures for the development of a model and external validation on a different population (TRIPOD level 3), both models were included in our analysis. If a performance measure was presented separately for a model including only a PRS and the same model combining the PRS with genetic and non-genetic risk factors, both models were considered separately. The same method was performed if a model presented results for different subtypes of BC or different ethnicities. However, if a study presented different performance measures for the PRS and some genetic and non-genetic risk factors individually, only the most comprehensive model was retained.

We also presented the calibration assessed with the Hosmer–Lemeshow test or the expected-to-observed (E/O) ratio, and the reclassification assessed with the net reclassification index (NRI) when available. The Hosmer–Lemeshow test provides a chi-square and *p*-value that indicates the goodness of fit. When this test is not statistically significant, it indicates a lack of evidence of model miscalibration. The expected-to-observed (E/O) ratio provides a ratio of the total expected number of cases (individuals with the outcome) to the observed number of cases. A value of 1 indicates that the model is perfectly calibrated, while values less than 1 and above 1 indicate, respectively, that the model is either underpredicting or overpredicting the total number of cases in the population [31]. The NRI seeks to quantify the agreement between risk classification and event status (cases and controls) when comparing an old model to a new model given a set of predefined risk categories. It allows evaluating the incremental value in the predictive capacity of new predictors to an existing set of predictors. The statistic is calculated as follows *P*(up|case) − *P*(down|case) + *P*(down|control − *P*(up|control), and its value ranges between −2 and 2. The terms “up” and “down”, respectively, refer to a new risk model placing an individual into a higher risk category or a lower risk category compared to the old model [33,34].

Finally, we performed sensitivity analyses by using multiple comparative assessments based on characteristics such as the populations on which the model was developed, the number of SNPs, the type of risk prediction tools used, the BC subtype and the age category to determine their impact on the models’ performance.

### 2.6. Risk of Bias of Individual Studies

We used PROBAST Prediction model Risk Of Bias Assessment Tool) [35], a tool which is organized into four domains (participants, predictors, outcome and analysis) to assess the risk of bias of individual studies that developed or validated multivariable diagnosis or prognosis prediction models. We used the same classification as the tool to indicate whether the studies were at low (+), high (−) or unclear (?) risk of bias for each domain separately. Based on our classification for each domain, we followed the PROBAST’s method [35] to determine the overall risk of bias for a study.

## 3. Results

### 3.1. Study Selection

**Figure 1:**
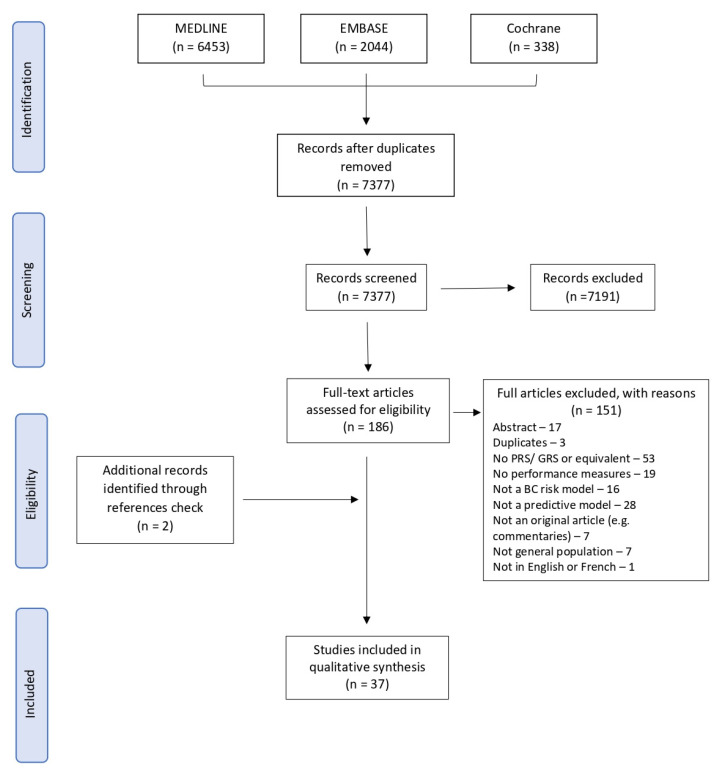
PRISMA flowchart of exclusion criteria.

A total of 7377 records were found from our search strategy after removing duplicates. We excluded 7191 records after screening their titles and abstracts and assessed the full text of 186 papers. A flow diagram of the selection process is presented in Figure 1. At the full-text level, the main reasons for exclusion were that the studies did not include a PRS or a GRS (n = 53) and did not present a predictive model (n = 28) or a measure of performance (n = 19). Additionally, seven studies did not present a risk prediction model for the general population as two of them were developed on a population of working nurses [36,37], and five were developed either for women at increased familial risk of BC [38,39], with a previous diagnosis of BC [40,41] or with known genetic mutations [42]. Two additional studies were identified via reference screening of the included studies. In total, we included 37 studies [12,23,24,25,43,44,45,46,47,48,49,50,51,52,53,54,55,56,57,58,59,60,61,62,63,64,65,66,67,68,69,70,71,72,73,74,75] in our systematic review, presenting seven different risk prediction tools.

### 3.2. Characteristics of Included Studies

The main characteristics of individual studies are presented in Table 1. Studies included in this review presented the risk for different types of BC. Most predicted the risk of developing overall BC (n = 25) [12,24,25,43,44,45,46,49,51,54,56,58,61,62,63,64,65,66,67,69,70,71,72,74,75] or invasive BC (n = 11) [47,48,50,52,53,55,57,59,63,68,73], and BC subtypes such as ER-positive (n = 11) [7,12,45,47,48,49,50,52,57,67,70] and ER-negative (n = 10) [12,45,47,48,49,50,52,57,67,70]. Most studies were conducted in the United States (n = 7) [7,43,44,57,59,61,74], the United Kingdom (n = 5) [25,45,62,69,75], Sweden (n = 3) [23,46,66] and Australia (n = 3) [47,48,73]. Seven studies were conducted in Asian countries [54,65,70], including four in multiple countries [50,64,68,72]. Of our 37 studies, 8 (21.6%) [12,49,50,56,64,67,68,74] presented models with only genetic factors. The number of SNPs used to calculate the PRS varied between 7 [57] and 5218 [74]. Most studies selected SNPs based on previously identified SNPs from published studies. Others selected SNPs associated with BC at a predefined threshold, level of significance or associated specifically with a certain ancestry group. The most common development method of the PRS was based on the cumulative effect of the per-allele odds ratio and the number of risk alleles [57]. However, newer methods such as Bayesian approaches [7,59] or risk prediction algorithms [72] were also used. Nineteen studies presented only the development of a risk model [7,43,46,47,48,49,52,54,55,56,57,59,62,63,64,65,66,67,71], and fourteen presented only its validation [23,24,25,44,45,50,51,53,58,61,69,73,74,75]. Nine included a method of internal validation [49,52,56,57,59,60,65,67,71], and four studies externally validated their model [12,68,70,72]. As for studies combining genetic and non-genetic risk factors, the selection of non-genetic risk factors was mostly based on those included in previously validated prediction tools such as the BCRAT [43,44,45,46,47,48,52,53,54,57,58,61] or IBIS [25,43,45,48,53,58,62,69,73]. Studies sample size ranged from 39 to 33,673 cases and 51 to 286,801 controls (Supplemental Table S2). Twelve studies used logistic regression to create the final model combining the PRS and genetic and non-genetic risk factors [7,43,44,45,52,55,59,63,66,69,70,75], and two used Cox regression [54,71].

### 3.3. Characteristics of Risk Prediction Models

Table 2 shows the main non-genetic predictors used in combined models. The number of predictors included in the models varied between one [75] and twenty-five [58]. Almost all models used the current age of women as a predictor of BC. Age was considered either by directly introducing it in the models as an independent variable or by stratifying by age groups [25,47,51,58,73]. Age at menarche, age at menopause, age at first live birth and family history of BC were also used in the majority of models. Details on participants and risk models are shown in Supplementary Table S2. Most models (55.0%) were developed or validated in populations of European descent, 26.3% in Asian populations, 12.5% in populations of African descent and 6.3% in Hispanic populations. A little more than 55%, 30% and 15% of the models estimated the 5-year, 10-year and lifetime risk of developing BC, respectively. In addition, one model estimated the 2-year [66] and 3-year [71] BC risk. Among the models based on a combination of genetic and non-genetic risk factors, 32.7% were an upgraded version of the BCRAT, 21.2% of the IBIS, 9.6% of the Breast Cancer Surveillance Consortium (BCSC) and 7.7% on the BOADICEA tool.

### 3.4. Discriminatory Accuracy

**Figure 2:**
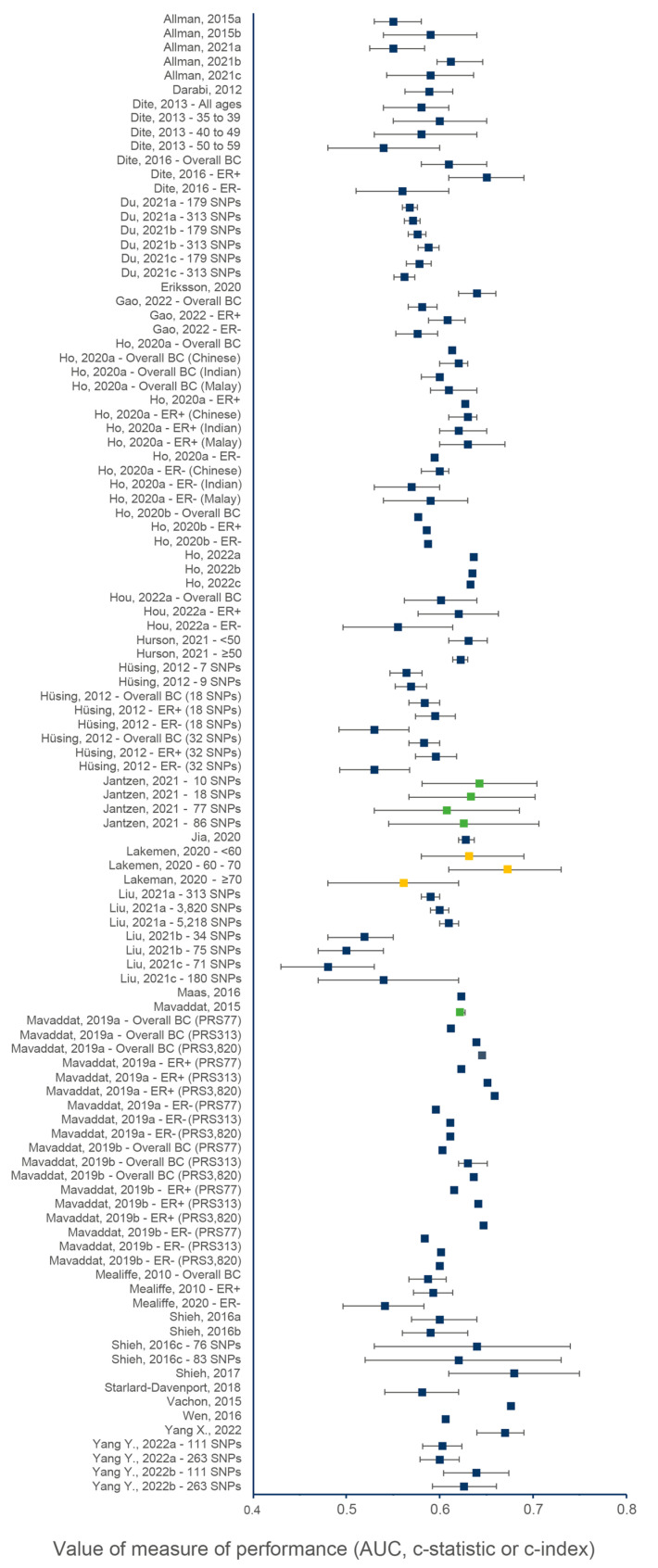
Discriminative performance of individual risk models, including only a PRS. Each dot represents a measure of discrimination for different versions (represented by the letters when applicable) of risk models as described in Supplementary Table S2. The horizontal segment represents the 95% CIs when provided. Blue, green and yellow dots indicate that the AUC, c-statistic and c-index were the measure of performance, respectively.

**Figure 3:**
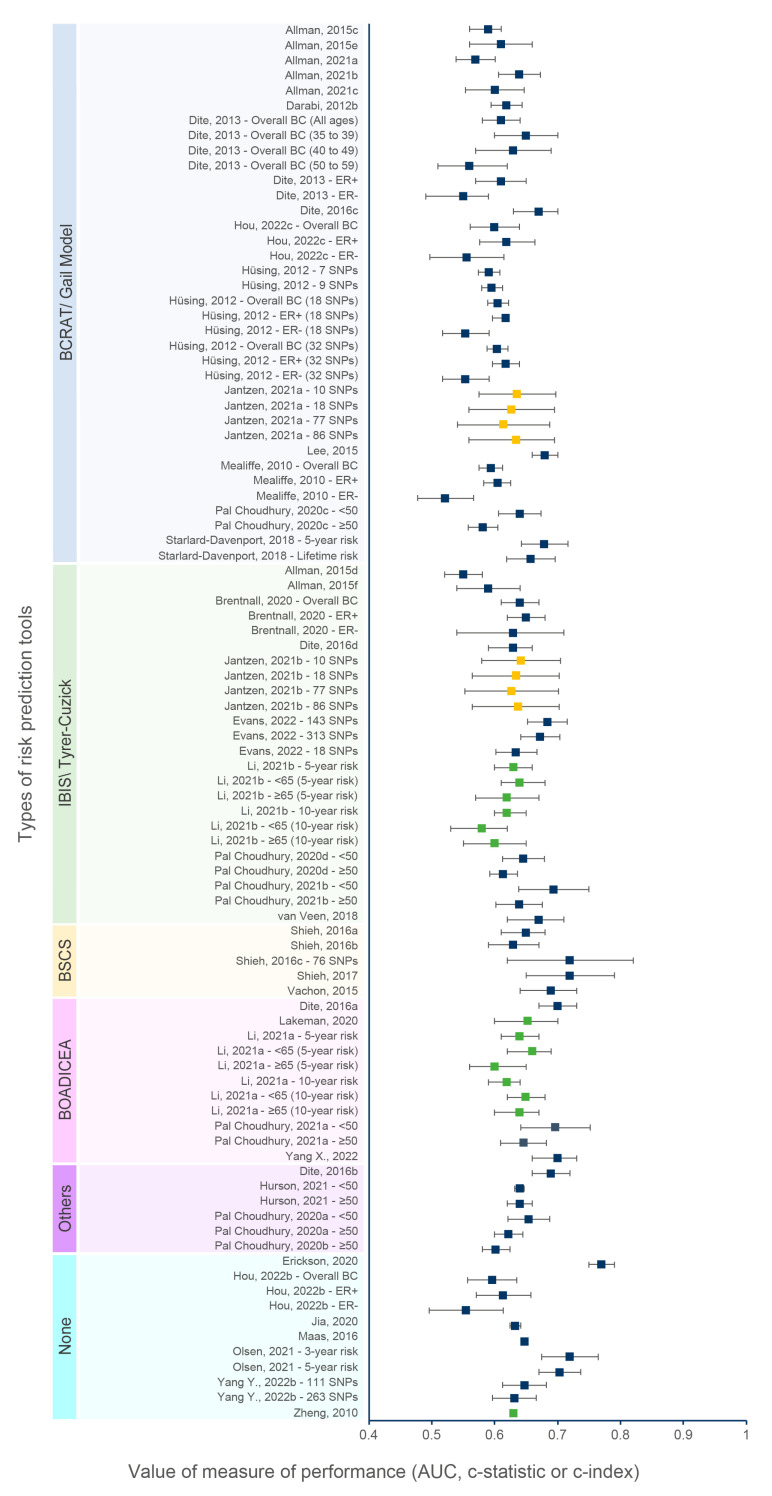
Discriminative performance of individual risk tools for models including a PRS and genetic and non-genetic risk factors. Each dot represents a measure of discrimination for different versions (represented by the letters when applicable) of risk models as described in Supplementary Table S2. The horizontal segment represents the 95% CIs when provided. Blue, green and yellow dots indicate that the AUC, c-statistic and c-index were the measure of performance, respectively.

Figure 2 and Figure 3 show the discriminative performance of the individual risk models and their 95% confidence intervals (CI) when provided. For the models including a PRS only, 105 measures of discrimination were reported representing different versions of certain models (Supplemental Table S2). The discrimination measure values ranged from 0.48 (95% CI = 0.43–0.53) [74] to 0.68 (95% CI = 0.61–0.75) [7]. For models including a combination of genetic and non-genetic factors, a total of 93 measures of discrimination were also reported. The discrimination measure values ranged from 0.52 (95% CI = 0.48–0.57) [57] to 0.77 (95% CI = 0.75–0.79) [66]. The model by Shieh et al. (2017) [7], including only a PRS, had the best predictive capacity and predicted the 5-year ER-positive risk in women of European descent, whereas the one by Liu et al. (2021) [74] had the lowest predictive capacity and predicted the 5-year overall BC risk in women of Latinx descent. The combined model by Erikson et al. (2020) [66] had the best predictive capacity and predicted the 2-year overall BC risk in women of European descent, and the combined model by Mealiffe et al. (2010) [57] had the lowest predictive capacity and estimated the 5-year risk of ER-negative BC in women of European descent. Due to differences in outcomes, predictors and time frame, direct comparisons between models could not be made; thus, these results are intended only to provide an overview of the models’ performance.

### 3.5. Calibration Accuracy

Of the 37 studies, 20 (54.1%) provided a measure of calibration for their models (details shown in Supplementary Table S3) [12,23,24,25,43,45,46,47,48,51,53,57,58,59,61,62,63,70,71,73]. Most studies used the Hosmer–Lemeshow test or the expected-to-observed (E/O) ratio. A few studies also provided a calibration slope or plots. When looking at the Hosmer–Lemeshow test, most models had *p*-values greater than 0.05, indicating no evidence of the data not fitting the models. Indeed, since the Hosmer–Lemeshow test is often underpowered, a *p*-value greater than a threshold should not by itself justify good calibration. On the other hand, the E/O ratio had values ranging from 0.73 (95% CI: 0.63–0.85) [73] to 1.23 (95% CI: 0.18–2.28) [70], indicating that models were either underpredicting or overpredicting the risk of BC.

### 3.6. Net Reclassification Improvement

A measure of reclassification was provided in twelve studies [23,43,46,47,48,51,52,57,61,63,65,71]. All but three studies [23,51,65] used the NRI to quantify the discriminatory ability of the combined models with a PRS and genetic and non-genetic risk factors compared to the same model without the PRS. Studies usually used a net reclassification measure for cases (identified as events—NRI_e_) and controls (identified as nonevents–NRI_ne_) at a predefined risk threshold. NRI values ranged from −0.029 (*p*-value = 0.5) [47] to 0.181 (95% CI 0.09–0.27) [43]. In general, the addition of the PRS to clinical risk prediction tools such as the BCRAT or IBIS improved the classification of patients, with cases going into higher risk categories and controls into lower risk categories.

### 3.7. Sensitivity Analysis

#### 3.7.1. Effect of Number of SNPs

The number of SNPs included in a PRS seemed to have a modest positive impact on the predictive capacity of risk prediction models. Based on the studies that developed multiple PRS with different numbers of SNPs [12,49,52,53,68,69,72,75], the predictive capacity of the PRS increased only with a considerable increase in the number of SNPs. Otherwise, there was no statistically significant change in performance. Husing et al. (2012) [52] developed a PRS on women of European descent with 7, 9, 18 and 32 SNPs, and their AUCs were 0.56 (95% CI: 0.55–0.58), 0.57 (95% CI: 0.55–0.59), 0.58 (95% CI: 0.57–0.60) 0.58 (95% CI: 0.57–0.60), respectively. Mavaddat et al. (2019) [12] validated a PRS with 77, 313 and 3820 SNPs for the risk of overall BC in White women, and their AUCs were 0.61, 0.64 and 0.65, respectively. Similarly, Liu et al. (2021) [74] validated three PRS developed in women of European descent with 313, 3820 and 5218 SNPs, two PRS developed in women of Hispanic descent with 71 and 180 SNPs and two PRS developed in women of African descent with 34 and 75 SNPs. The PRS with 313, 3820 and 5218 SNPs had respective AUCs of 0.59 (95% CI: 0.58–0.60), 0.60 (95% CI: 0.59–0.61) and 0.61 (95%CI: 0.60–0.62), whereas the PRS with 71 and 180 SNPs had respective AUCs of 0.48 (95% CI: 0.43–0.53) and 0.54 (95% CI: 0.47–0.62). However, for the PRS developed on women of African descent, the predictive performance decreased with an increasing number of SNPs. The AUC was 0.52 (95% CI: 0.48–0.55) for 34 SNPs and 0.50 (95% CI: 0.47–0.54) for 74 SNPs. The study by Jantzen et al. (2021) [53] showed similar results with a PRS developed on White women with 10, 18, 77 and 86 SNPs. Their respective c-index values were 0.643 (95% CI: 0.581–0.704), 0.634 (0.567–0.702), 0.608 (95% CI: 0.530–0.685) and 0.626 (95% CI: 0.545–0.706).

#### 3.7.2. Effect of Age

Five studies stratified their models by age groups and presented a model for younger women (usually less than 50 years old) and one for older women (usually 50 years and older) [25,47,51,58,73]. Three studies [25,51,58] used the PRS developed by Mavaddat et al. (2019) [12] with 313 SNPs and predicted the 5-year risk of overall BC in White women and one [73] used the same PRS but predicted both the 5- and 10-year risk of invasive BC in White women. Risk prediction tools tended to perform slightly better on younger women, although differences were not statistically significant. Studies by Hurson et al. (2021) [51] and Pal Choudhury et al. (2020) [58] used the iCare-Lit tool to validate their combined models. Their respective AUCs for women younger than 50 were 0.64 (95% CI: 0.62–0.66) and 0.65 (95% CI: 0.62–0.69). The corresponding values were 0.64 (95% CI: 0.63–0.65) and 0.62 (95% CI: 0.60–0.65) for those 50 years and older. Another study by Pal Choudhury et al. (2021) [25] using the BOADICEA tool yielded similar results. The predictive performance (AUC) for women younger than 50 years old was 0.70 (95% CI: 0.64–0.75), and for women 50 years and older, it was 0.65 (95% CI: 0.61–0.68). Lastly, the study by Li et al. (2021) [73] validated the 5-year risk using the BOADICEA and IBIS tools. The c-statistic for women younger than 65 years old were 0.66 (95% CI: 0.62–0.69) and 0.64 (95% CI: 0.61–0.68) and for women 65 years and older, 0.60 (95% CI: 0.56–0.65) and 0.62 (95% CI: 0.57–0.67) for each tool, respectively.

#### 3.7.3. Effect of Combining a PRS and Genetic and Non-Genetic Risk Factors

Many studies reported results for a model including a PRS only and models including the same PRS combined with genetic and non-genetic risk factors [23,43,44,46,47,48,51,52,53,55,57,59,60,61,72,75]. When comparing the same models with and without the addition of risk factors to the PRS, we observed that the combination of genetic and non-genetic risk factors and a PRS improved the discriminative capacity. The model with the greatest improvement from the combination of a PRS and genetic and non-genetic risk factors is the one by Dite et al. (2016) [48]. This study, based on the BOADICEA tool, estimated the 5-year risk and presented discrimination measures for the clinical risk factors only (AUC: 0.66; 95% CI: 0.63–0.70), for the PRS only (AUC: 0.61; 95% CI: 0.58–0.65) and for the combined model (AUC: 0.70; 95% CI: 0.67–0.73). The same study also used the BRCAPRO tool, and the AUC for clinical risk factors only was 0.65 (95% CI: 0.62–0.68) and 0.69 (95% CI: 0.66–0.72) for the combined model. Another study by Shieh et al. (2016) [59] based on the BSCS tool estimating the 5-year BC risk on women of East Asian descent showed great improvement from the addition of a PRS with an AUC of 0.72 (95% CI: 0.62–0.82) for the combined model compared to 0.64 (95% CI: 0.53–0.74) for the PRS only. One of the most comprehensive models is the one by Yang X. et al. (2022) [23], combining a PRS and genetic and non-genetic risk factors using the BOADICEA tool. Their PRS-only model had an AUC of 0.67 (CI 95%: 0.64–0.69), whereas the addition of genetic and non-genetic risk factors, including information on family history, risk factors such as lifestyle, hormonal and reproductive risk factors, mammographic density and pathogenic variants in BC susceptible genes such as *BRCA1* and *BRCA2* provided an AUC of 0.70 (95% CI = 0.66–0.73).

#### 3.7.4. Effect of Ethnicity

In general, models developed on populations of European descent performed better than models developed on populations from other ethnicities. A study by Allman et al. (2021) [44] validated the same model on three different populations: African Americans, Whites and Hispanics. The AUCs were 0.57 (95% CI: 0.54–0.60), 0.64 (95% CI: 0.61–0.67) and 0.60 (95% CI: 0.55–0.65), respectively. Liu et al. (2021) [74] also validated three PRS originally developed on women of European descent in women of African and Latinx descent. The AUCs were consistently lower in women of African and Latinx descent. One PRS had AUCs of 0.60 (95% CI: 0.59–0.61), 0.55 (95% CI: 0.51–0.58) and 0.55 (95% CI: 0.50–0.60) in women of European, African and Latinx descent, respectively. However, models developed on populations of Asian descent tended to have similar predictive performance to those from populations of European descent. Ho et al. (2020) [50] validated the PRS developed by Mavaddat et al. (2019) [12] on an Asian population, which had been previously validated on a White population. Both AUCs were close, with values of 0.61 and 0.63. A more recent study by Ho et al. (2022) [68] showed that a PRS developed on a population of Asian descent using a Bayesian polygenic prediction approach and a combination of European and Asian-specific SNP weights from a subset of SNPs by Mavaddat et al. (2019) [12] provided an AUC of 0.64. In fact, PRS based on SNPs associated with BC among a specific ethnicity performed better than general PRS. For example, Shieh et al. (2016) [59] applied to an East Asian population a PRS with 76 SNPs associated with BC in that subpopulation and a general PRS with 83 SNPs associated with European descent populations. The Asian-specific PRS had an AUC of 0.64 (95% CI: 0.53–0.74), whereas the general PRS in East Asians had an AUC of 0.62 (95% CI: 0.52–0.73).

#### 3.7.5. Effect of Prediction Time Frame

Most models predicted the risk of BC within 5 or 10 years. Some models also predicted the lifetime risk (i.e., until the age of 80 to 90). Generally, models with a shorter prediction time frame had better predictive performances than models with a longer prediction time frame. Two studies used the same model on the same population but varied the prediction time frame [61,71]. The study by Starlard-Davenport et al. (2018) [61] predicted the 5-year risk and the lifetime risk of BC in African American women. The 5-year risk model had a slightly better performance than the lifetime risk model with an AUC of 0.68 (95% CI: 0.64–0.72) compared to 0.66 (95% CI: 0.62–0.70). We observed a similar pattern of results for the study by Olsen et al. (2021) [71], predicting the 5-year and 3-year risk with AUCs of 0.70 (95% CI: 0.67–0.74) and 0.72 (95% CI: 0.68–0.77), respectively.

#### 3.7.6. Effect of the Type of Risk Prediction Tools

Several risk prediction tools were upgraded with the addition of a PRS to genetic and non-genetic risk factors. Indeed, many studies were based on the BCRAT (Gail model) [43,44,46,47,48,52,53,54,57,58,61,70], IBIS (Tyrer–Cuzick) [25,43,45,48,53,58,62,69,73], BOADICEA [23,24,25,48,73], BRCAPRO [48], iCARE-Lit [51,58] and BCSC [59,60,63] tools. For instance, Allman et al. (2021) [44] used a streamlined version of the Gail tool based only on risk factors such as age and family history that could be easily used by physicians in the absence of the complete information required by the tool. Models based on the BOADICEA tool usually performed well, considering that it is the most comprehensive BC prediction risk tool in terms of risk factors included. The value of their predictive statistic ranged from 0.62 (95% CI: 0.59–0.64) to 0.70 (0.66–0.73). Several studies compared the predictive capacity of different tools on the same population [25,43,48,53,58,73]. The predictive values of different tools tended to be similar when validated on the same population. Jantzen et al. (2020) [53] used the BCRAT and IBIS model with a PRS of 86 SNPs to evaluate the 5-year BC risk on White women and had the same c-index value of 0.63 (95% CI: 0.56–0.70). This observation was similar to the study by Allman et al. (2015) [43] comparing the same two tools but on African Americans and Hispanics. For African Americans, the respective AUCs for the BCRAT and IBIS tools were 0.59 (95% CI: 0.56–0.61) and 0.55 (95% CI: 0.52–0.58), whereas for Hispanics, they were 0.61 (95% CI: 0.56–0.66) and 0.59 (0.54–0.64). Another study by Dite et al. (2016) [48] predicting the 5-year risk of invasive BC on White women compared four tools, namely the BOADICEA, BRCAPRO, BCRAT and IBIS tools, and obtained AUCs of 0.70 (95% CI: 0.67–0.73), 0.69 (95% CI: 0.66–0.73), 0.67 (95% CI: 0.63–0.70) and 0.63 (95% CI: 0.59–0.66), respectively.

#### 3.7.7. Effect of Breast Cancer Subtypes

Some articles presented discrimination measures for overall BC risk and different subtypes, usually estrogen-positive (ER+) and estrogen-negative (ER-) [12,45,47,48,49,50,57,67,70]. Three studies used the overall BC PRS within subtypes [47,48], one of which also constructed a subtype specific PRS using corresponding BC cases [70]. Four other studies used the same set of SNPs as their overall BC PRS but assigned ER subtype-specific weights to the SNPs [12,49,50,57]. Two other studies used a subset of SNPs associated with each ER subtype that achieved genome-wide significance at a predefined *p*-value [45,67]. Additionally, two studies presented some results for progesterone-positive (PR+) and progesterone-negative (PR-) BC (results not shown) [48,52]. Mavaddat et al. (2019) [12] validated a 313-SNP PRS to predict the 10-year risk of developing overall BC, ER+ and ER-. The respective AUCs were 0.64, 0.65 and 0.61. Du et al. (2021) [49] used the PRS developed by Mavaddat et al. (2019) [12] to predict the lifetime risk of overall BC, ER+ and ER- in women of African descent and obtained AUCs of 0.58 (95% CI: 0.57–0.60), 0.59 (95% CI: 0.58–0.60) and 0.56 (95% CI: 0.55–0.57), respectively. Ho et al. (2020) [50] evaluated the 10-year risk using a 287-SNP PRS for Asian women and obtained AUCs for overall, ER+ and ER- BC of 0.61, 0.63 and 0.59, respectively. On the other hand, Brentnall et al. (2020) [45] added a 143-SNP PRS to the Tyrer–Cuzick tool. When stratifying their results by BC subtypes, the AUC for overall BC risk was 0.64 (95% CI: 0.61–0.67), 0.65 (95% CI: 0.62–0.68) for ER+ and 0.63 (95% CI: 0.54–0.71) for ER-. The models’ discriminating capacity seemed to be lower for ER- BC subtypes.

### 3.8. Quality of Reporting

The TRIPOD checklist considers 22 items to be essential for good reporting of studies developing or validating multivariable prediction models [30]. Of the 37 studies, only four encompassed all the items on the checklist. The vast majority of studies did not follow the title’s recommendation. Namely, they did not identify if the study was either a development or a validation model. In the methods, the description of how the missing data were handled was the most omitted item. For most studies, the results section was clear and complete. However, seven studies did not report confidence intervals for all discriminative measures [12,50,55,63,64,65,68]. Also, as recommended by the TRIPOD guidelines, calibration performance should be included in all prediction models, but it was assessed less often than discriminative performance. All studies reported their limitations and provided an overall interpretation of their results given those limitations. Finally, other information, such as supplementary materials, was often provided, and a funding statement was present in all studies.

### 3.9. Risk of Bias within Studies

**Figure 4:**
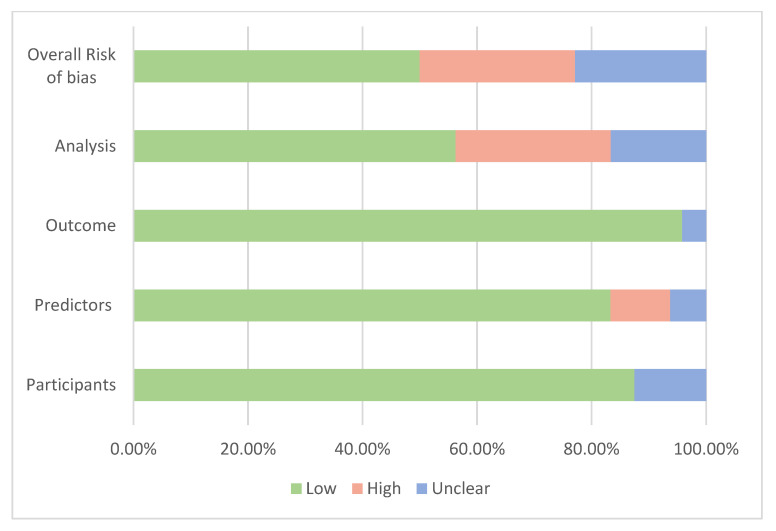
Summary of the assessment of risk of bias.

Assessment of the risk of bias is presented in Figure 4 based on the four domains of the PROBAST tool [35]. Overall, 19 studies were at low risk of bias, 7 were at high risk and 11 were at unknown risk of bias. When the participant domain was at unclear risk, it was mostly because participants’ inclusion or exclusion criteria were not described or not described with enough details to determine if they were appropriate. For the predictors’ domain, the main reason for the high or unclear risk of bias was the absence of important predictors such as age when the model was developed or validated. A couple of studies were concerning for the outcome domain since it was unclear whether the outcome was a preclinical stage of cancer [66,71]. Most risks of bias occurred in the analysis domain as many development models did not account for complexities in the data, such as competing risk or model overfitting, underfitting and optimism, or did not explain how they handled missing data. These risks of bias, such as the potential overfitting, were mentioned in some studies [64,68,72].

## 4. Discussion

The goal of this systematic review was to appraise and critically assess different prediction models incorporating a PRS used to estimate the risk of developing BC for women in the general population. We identified 37 studies, of which 8 included genetic factors only, whereas the rest combined genetic and non-genetic risk factors. The combined models were based on 7 different risk prediction tools and provided 93 measures of discrimination. For models’ development, the median value of discriminative performance measures was 0.60 (range = 0.53 to 0.68) for models with PRS only and 0.62 (range = 0.52 to 0.77) for models combining PRS and genetic and non-genetic risk factors. For the models’ validation, the median value of discriminative performance measures was 0.61 (range = 0.48 to 0.67) for models with PRS only and 0.64 (range = 0.55 to 0.70) for models combing PRS and genetic and non-genetic risk factors. Although the increase in AUC from the combination of the PRS and genetic and non-genetic risk factors may look small, from a public health perspective, even a modest increase in discriminative performance may lead to a considerable improvement in overall risk stratification levels and be clinically meaningful [23].

Comprehensive BC risk prediction tools incorporating known risk factors could have two potential applications. They can be used as risk-stratification tools to improve the ability to identify women in the general population at increased risk who would most likely benefit from personalized screening recommendations. They can also be used as risk prediction tools to predict the risk of developing overall BC and molecular subtypes in healthy women. However, there are many aspects to consider when evaluating if these tools could be part of clinical routine or public health practices for risk prediction and stratification. The first is to determine the models’ capacity to predict the outcome of interest in a defined population, known as the analytical validity [8]. The second is to evaluate the clinical utility of the tools (i.e., their usefulness, benefits and harms) [8]. The first aspect may be taken into account by evaluating, as performed in this review, the discriminating capacity, the calibration or the fit of a model and, additionally, other performance measures such as the net reclassification index that has been proposed as an alternative or adjunct to discrimination and calibration measures [18].

A first consideration when assessing the predictive performance of a model is that a risk prediction model should be developed in one sample of a data set and validated in a separate independent sample or new data [30]. In fact, associations between risk factors and BC derived from the same data set in which the model was developed may occur by chance due to multiple testing. This problem becomes important with a relatively small sample size with many risk factors included in the model. In studies with small sample size, there is a serious risk of selecting unimportant variables and omitting some variables relevant to the model [86]. At the same time, studies with a very large sample size are more likely to include statistically significant variables but with little clinical importance [87]. Simulation studies have suggested that the ideal number of subjects with events should be at least 10 and safer with 20 or more per risk factor in order to build a valid model [88,89]. As per the results of our review, the number of variables included in the models varied from 2 to 25 variables, so the required number of BC cases should range between 20 and 500 subjects. In this regard, two models could present issues. The model estimating the 10-year ER-negative BC risk by Brentnall et al. (2020) [45] with an AUC of 0.63 (95% CI: 0.54–0.71) had 39 cases for 7 predictors, including the PRS score. The same situation is also present with the model by Shieh et al. (2016) [59] with an AUC of 0.72 (95% CI: 0.65–0.79) but 51 cases for 7 predictors, including the PRS score. Therefore, these models could lead to overoptimistic results in the validation data [90]. However, robust methods can facilitate variable selection, especially when there is a large number of predictors, and can account for many challenges in SNP selection and model specification [91]. Thus, some studies have used more sophisticated methods to select or develop their PRS, such as penalized regression or Bayesian approach [12,67,68,70,72] and show promising results, particularly in diverse populations.

As models perform better in the sample in which they were developed rather than in a different sample or a completely new population, model development should include a validation process. However, about a third of the models did not present any validation (i.e., internal or external validation), which brings concerns regarding the validity of some models as they might not be ready to be used. Nonetheless, several studies were a validation or an extension of existing prediction models. While the addition of a PRS to existing models increased their discriminative accuracy, the number of SNPs considered in the PRS varied widely from one study to another. It could be another factor influencing their predictive performance. SNPs included in a PRS should be inherited independently (i.e., in linkage equilibrium). Some studies excluded SNPs in high linkage disequilibrium from those reported in the original study [75] or used them as proxies for risk variants not available in their dataset [52,59,75]. With the discovery of more SNPs from larger GWAS, the AUC of the risk prediction models is improving. For instance, the oldest model by Mealiffe et al. (2010) [57] had an AUC of 0.58 (95% CI: 0.57–0.60) for the prediction of overall BC using only a 7 SNP-PRS while a more recent model by Mavaddat et al. (2019) [12] had an AUC of 0.63 (95% CI: 0.63–0.65) for the prediction of overall BC using only a 313 SNP-PRS. Since a small improvement in AUC can have a significant impact on risk stratification, one relevant parameter to evaluate a PRS should be the proportion of the polygenic variance attributable to the PRS as expressed by the odds ratio per 1 standard deviation [14].

Another consideration when developing a risk prediction tool is to choose the timeframe for which risk should be predicted [18]. Follow-up time has been shown to have an impact on discriminative accuracy measures such as the concordance index and is likely important to understand differences in predictive ability [92,93]. In our review, the model with the highest discriminative accuracy had the shortest prediction time frame. In fact, the model by Eriksson et al. (2020) [66] had an AUC of 0.77 (CI 95%: 0.75–0.79) but estimated the 2-year BC risk, whereas most models predicted the 5-year, 10-year or lifetime risk. However, models evaluating short time frames would likely identify existing cancers or preclinical cases. The lead time for BC, which is the period between the early detection of BC by screening and the moment the cancer clinically presents or is diagnosed, is about two to three years [94]. Thus, models predicting two or three-year risk are more likely to be diagnostic tools and be considered as screening tests. Tools with longer timeframes could be more effective in predicting BC risk in a screening setting for risk stratification and be used as complements of early detection tools [66].

The choice of the risk prediction tool, especially in a public health setting, should also be examined [18]. In our review, based on the latest version of the risk prediction tools and considering the overall risk of bias and the population size, we observed that the best combined models were validation models derived from the BOADICEAv.6 and the Tyrer–Cuzick v.8 tools [23,25]. Both models predicted the 5-year risk of overall BC in White women and used the 313-SNP PRS developed by Mavaddat et al.(2019) [12]. Although both models have limitations, including that they are not as well-calibrated in non-White populations, the Tyrer–Cuzick tool for women over 50 provided an AUC of 0.69 (95% CI: 0.64–0.75), and the BOADICEA provided an AUC of 0.70 (95% CI: 0.66–0.73). The Tyrer–Cuzick tool used a wide range of non-genetic risk factors but was missing important risk factors such as breast density. The BOADICEA tool was the most comprehensive, combining genetic risk factors such as the PRS and pathogenic variants in BC susceptibility genes as well as non-genetic risk factors, including breast density.

Some tools included predictors easily collected in routine clinical practice or even by questionnaires like smoking status and BMI, whereas others include predictors requiring an extensive medical examination. For example, biopsy histopathology (i.e., the presence of atypical hyperplasia) increases the risk of a woman for BC and is included in the BCRAT model [95]. However, it can be difficult or expensive to collect and, therefore, has limited use in a population-wide public health approach. Indeed, most models that considered this predictor did not have the information and coded the variable as unknown [43,47,48,57,73]. On the other hand, including unique risk factors such as mammographic masses and microcalcifications, as performed by Eriksson et al. (2020) [66], could improve BC risk prediction tools. Lastly, the effect of some risk factors, such as family history, needs to be carefully considered as it may inflate the value of AUC when cases have enriched and strong family history of BC. This has been shown in the study by Starlard-Davenport et al. (2018), reporting an AUC of 0.68 (CI 95%: 0.64–0.72) for the 5-year risk of overall BC in African American women that was significantly higher than other models developed in African American women but unselected for family history [61].

The heterogeneity of populations is also challenging when choosing a prediction tool. We observed that only a limited number of risk models were developed or validated in non-White populations. The models developed or validated on individuals of Latin or African descent performed particularly poorly, with the highest AUC of 0.68 (CI 95%: 0.64–0.72) for African descent [61] and 0.60 (CI 95%: 0.55–0.68) for Latin descent [44]. In comparison, models developed or validated in Asian women showed fairly similar performance to the ones developed in White populations [50], with the highest AUC of 0.72 (CI 95%: 0.62–0.82) [59]. When integrating PRS into public health practice, we need to ensure that it does not exacerbate health disparities. Currently, risk prediction tools, including a PRS, are not easily generalizable across diverse populations. Other ethnic groups have been underrepresented in GWAS, including about 80% of participants from European ancestry [96,97]. This is even more problematic for African, Hispanic or Indigenous individuals who were included in less than 4.0% of the GWAS studies from the first decade of their development [98]. The poor performance of models for non-White populations may be explained by the fact that PRS are usually calculated as a weighted sum of the risk alleles of SNPs derived from GWAS. However, most PRS do not account for effect sizes being different than the reference of populations of European descent [98,99]. Also, events such as the “flip-flop” phenomenon where a variant is a risk factor in one population but a protector in another have been observed in about 30 to 40% of variants across studies and affect the performance of risk prediction models [11,78]. Thus, the performance of the PRS declines with increasing genetic divergence from the reference population, resulting in attenuated associations partly due to variation in linkage disequilibrium patterns and allele frequencies. Therefore, many researchers and organizations have raised the need for more diverse biobanks to conduct GWAS [100].

In conclusion, although the addition of new risk factors such as SNPs has improved the discriminative ability of risk prediction models, they still need to be further evaluated to address the potential barriers to using these tools and the appropriate threshold for interventions and/or recommendations [17,18]. Also, there is currently no recommendation for any tools predicting individual risk to be used as the standard in a screening context. Therefore, comparative assessment and validation of risk prediction models in the same populations would be necessary to evaluate the effect of individual risk factors and determine which tool could be useful at the population level [25,101].

## 5. Strengths and Limitations

Our review has many strengths worth mentioning. To our knowledge, this is the first systematic review focusing specifically on BC risk prediction models, including a PRS. We used a rigorous methodologic approach where two reviewers independently performed each step of study selection and data extraction. The publication of the review protocol was another strength that ensured transparency in our work. Finally, including multiple measures of discrimination from the same study allowed us to appraise the incremental improvement resulting from adding genetic and non-genetic factors to the PRS. However, our review also has some limitations. Although two reviewers were involved in identifying the studies, we cannot exclude the possibility that some studies might have been missed. In fact, genetic research related to cancer is a growing field, and new articles on the subject are published regularly. We included only studies published in English or French, and those available in the gray literature or published in other languages were not considered. Also, we did not include studies focusing on the development or validation of new methodological methods for risk prediction tools or simulation studies, as those were outside the scope of this review. Furthermore, it was not possible to present pooled results of individual studies in a meta-analysis due to the heterogeneity of included studies in terms of predictors and PRS. Some limitations were due to the quality of individual studies. A measure of calibration or reclassification was not always provided in models, making it difficult to determine how close the estimated risk was to the predicted risk. Finally, studies showed the predictive performance of models, but only some of them considered the clinical and practical utility of these models [102].

## 6. Conclusions

Our research brings evidence on BC risk prediction tools incorporating a PRS. This review shows that the combination of genetic and non-genetic risk factors and PRS tends to increase the predictive performance compared to the PRS only and can improve risk stratification in the population. While most tools’ discriminative accuracy was still modest, predictive performance is only one component when considering if a risk prediction tool will be implemented and useful in a clinical or public health setting. Many barriers, legal, social, ethical and economic, can influence the implementation of a prediction tool. Therefore, this review is only a first step in understanding the issues related to the validation of BC risk prediction tools, including a genetic risk score, and more studies are needed to shed light on potential challenges in implementing these tools.

## Figures and Tables

**Table 1:** Characteristics of individual studies based on the type of included risk models.

Author, Year	Country	Type of BC	Number of SNPs	Selection of SNPs	Method of Development of GRS or PRS	Selection of Risk Factors	Method of Development of Combined Model	TRIPOD Level
Models based on genetic risk factors alone								
Du, 2021 [49]	Various (USA, Ghana, Nigeria and Barbados)	Overall BC, ER-positive, ER-negative	179 and 313 SNPs	SNPs that reached genome-wide statistical significance in GWAS analyses and SNPs from a previously published study [12].	Cumulative effect at a risk locus of the weights of each SNP multiplied by the risk allele dosage of each SNP.	__	__	2a
Gao, 2022 [67]	Various (Barbados, Ghana, USA)	Overall BC, ER-positive, ER-negative	307 SNPs	Modified version of the hard-thresholding based on stepwise forward logistic regression outlined by Mavaddat et al. [12].	Three methods were evaluated: (1) Cumulative effect of the per-allele log OR for BC associated with each SNP multiplied by the allele dosage for each SNP from genome-wide data in women of African Ancestry, (2) the 313 SNPs PRS using the effect size from Mavaddat et al. [12] and (3) the joint and hybrid PRS as a weighted linear combination of the two previous PRS (adapted method from Márquez-Luna et al. [76]).	__	__	2a
Ho, 2020 [50]	Various (China, Malaysia, India)	Invasive BC, ER-positive, ER-negative	287 SNPs	Available race-specific derived SNPs from Mavaddat et al. [12] based on an imputation accuracy score.	Cumulative effect of the weights of each SNP multiplied by the dosage of risk allele for each SNP.	__	__	4
Ho, 2022 [68]	Various (China, India, Taiwan, Singapore, Korea, Japan, Malaysia)	Invasive BC	46, 287 and 2985 SNPs	Multiple approaches: (1) Clumping and threshold approach, (2) lasso penalized regression, (3) linear combination of European and Asian PRS, (4) integration of Asian weights into European PRS, (5) Bayesian polygenic prediction approach and (6) South Asian specific SNPs.	Cumulative effect of the weights of each SNP multiplied by the risk allele dosage of each SNP.	__	__	3
Liu, 2021 [74]	USA	Overall BC	313, 3820 or 5218 SNPs (European ancestry), 34 or 75 SNPs (African ancestry), 71 or 180 SNPs (Latinx ancestry)	Previously identified SNPs from published studies (2 studies in women of European ancestry [12,77], 2 in women of African ancestry [43,78] and 2 in women from Latinx ancestry [43,79].	Weighted sum of each variant effect size using the PLINK version 1.9 [80] to reconstruct seven previously developed and tested PRS with European, African and Latinx ancestries. Ambiguous variants, variants with allele mismatches, and variants with more than 3+ alleles from each PRS model were excluded.	__	__	4
Mavaddat, 2015 [56]	Various (Europe, Australia, USA)	Overall BC	77 SNPs	SNPs associated at *p* < 5 × 10^−8^ with overall or ER-negative BC by the COGS or previous publications.	Cumulative effect of the per-allele log OR multiplied by the number of alleles for the same SNP, ignoring departures from a multiplicative model.	__	__	2b
Mavaddat, 2019 [12]	Various (Europe, Australia, USA)	Overall BC, ER-positive, ER-negative	77, 313 and 3820 SNPs	Hard-thresholding based on stepwise forward regression that retained SNPs significantly association with overall or subtype-specific BC or lasso penalized regression.	For overall BC: Cumulative effect of the per-allele log OR for BC associated with each SNP and multiplied by the allele dosage for each SNP. For BC subtypes, four methods were evaluated: (1) using effect sizes for overall BC; (2) using effect sizes for subtype-specific BC; (3) using a hybrid method; (4) deriving subtypes-specific estimates using case-only ORs case-only ORs estimated by lasso combining with overall BC ORs.	__	__	3
Wen, 2016 [64]	Various (China, Japan, South Korea, Thailand, and Malaysia)	Overall BC	44 SNPs	SNPs associated with overall BC at *p* < 0.05 (one-sided).	Cumulative effect of the per-allele log OR for BC associated with the risk allele for each SNP.	__	__	1a
Models based on genetic risk factors and non-genetic risk factors								
Allman, 2015 [43]	USA	Overall BC	75 SNPs (African American), 71 SNPs (Hispanics)	Race-specific derived SNPs identified as being associated with BC from studies of White women [56] for which imputed genotypes were available.	Mealiffe et al. [57] approach. SNP-based relative risk score using ORs per-allele and risk allele frequency assuming independent and additive risks on the log-OR scale. Then, multiplying the adjusted risk values for each SNP.	From existing models (BCRAT and IBIS).	Log-transformed combined score (SNP-based score multiplied by model’s predicted 5-year risk) and age-adjusted using logistic regression.	1a
Allman, 2021 [44]	USA	Overall BC	77 SNPs (European ancestry), 75 SNPs (African American), 71 SNPs (Hispanics)	Race-specific derived SNPs from published GWAS.	Mealiffe et al. [57] approach. SNP-based relative risk score using ORs per-allele and risk allele frequency assuming independent and additive risks on the log-OR scale. Then, multiplying the adjusted risk values for each SNP.	From real-world clinical practice factors based on the BCRAT.	Log-transformed combined score (SNP-based score multiplied by model’s predicted 5-year risk) and age-adjusted using logistic regression.	4
Brentmall, 2020 [45]	UK	Overall BC (invasive or ductal carcinoma in situ), ER-positive, ER-negative	143 SNPs	Previously identified SNPs associated with BC from Michailidou et al. [10] and available in the dataset.	Multiplying the per-allele OR for each SNP, normalized by the average risk expected in the populations based on the assumed allele frequency.	From an existing model (Tyrer–Cuzick).	Regressing the PRS on adjustment factors (age, the natural logarithm of 10-year risk from the Tyrer–Cuzick model and mammographic density) in controls.	4
Darabi, 2012 [46]	Sweden	Overall BC	18 SNPs	Previously identified SNPs from Pashayan et al. [81].	Weighted average of effect estimates from separate studies obtained by the inverse variance method or multiplicative penetrance model for BC-associated SNPs.	From an existing model (Gail).	Method by Gail et al. [19] to estimate the probability of a woman with a particular risk profile developing BC in a specific time interval.	1a
Dite, 2013 [47]	Australia	Invasive BC, ER-positive, ER-negative	7 SNPs	Statistically significant SNPs associated with BC in GWAS and identified in the study by Mealiffe et al. [57]	Mealiffe et al. [57] approach. SNP-based relative risk score using ORs and risk allele frequency assuming independence of additive risks on the log-OR scale. Then, multiplying the adjusted risk values for each SNP.	From an existing model (BCRAT).	Multiplying the SNP risk score and the BCRAT risk score under the assumption of independence.	1a
Dite, 2016 [48]	Australia	Invasive BC, ER-positive, ER-negative	77 SNPs	Previously identified SNPs from Mavaddat et al. [56].	Mealiffe et al. approach [57]. SNP-based relative risk score using ORs per-allele and risk allele frequency assuming independence of additive risks on the log-OR scale. Then, multiplying the adjusted risk values for each SNP.	From existing models (BOADICEA, BRCAPRO, BCRAT, IBIS).	Multiplying the SNP-based score by model’s predicted 5-year absolute risk of BC (all risk factors were age-adjusted log 5-year risks).	1a
Eriksson, 2020 [66]	Sweden	Overall BC	313 SNPs	Previously identified SNPs from Mavaddat et al. [12].	Per-allele log OR for each SNP in a log-additive model.	Not specified—All considered were included.	Unconditional logistic regression stratified by age.	1a
Evans, 2022 [69]	UK	Overall BC (invasive or ductal carcinoma in situ)	18, 143 and 313 SNPs	Previously identified SNPs from two studies [45,63].	PRS143: Per-allele OR derived from published OR and allele frequency normalized around a relative risk of 1.0. PRS313: Cumulative effect of the log OR for each SNP multiplied by the corresponding number of minor alleles.	From existing model (Tyrer–Cuzick).	Regressing the PRS on adjustment factors.	4
Hou, 2022 [70]	China	Overall BC, ER-positive, ER-negative	24 SNPs	Previously identified SNPs from GWAS or meta-analyses found to be associated with BC risk in Chinese women.	Three different approaches, the first two based on the cumulative effect size, calculated as the per-allele log OR for BC associated with each SNP, multiplied by the number of effect alleles: (1) repeated logistic regression (RLR) (2) logistic ridge regression (LRR), (3) artificial neural network (ANN)-based approach.	Established BC risk factors.	Regressed the PRS against non-genetic risk factors or absolute risks predicted by the Gail-2 model.	3 *
Hurson, 2021 [51]	Various (Australia, Germany, Netherlands, Sweden, UK, USA)	Overall BC (In situ or invasive BC)	313 SNPs	Previously identified SNPs from Mavaddat et al. [12].	Cumulative effect of the per-allele log OR for BC associated with each SNP and multiplied by the allele dosage for each SNP.	From an existing model (iCARE-Lit).	Use of iCARE tool to incorporate risk factors and the PRS assuming a multiplicative joint association with disease risk, accounting for the correlation of PRSs with family history.	4
Husing, 2012 [52]	Various (USA, Europe)	Invasive BC, ER-positive, ER-negative	7, 9, 18 and 32 SNPs	Statistically significant SNPs in at least one GWAS at a genome-wide significance level (*p* < 10^−7^).	Log-additive model with individually weighted per-allele effects for each SNP.	Available factors from an existing model (BCRAT) selected using a backwards selection process.	Unconditional logistic regression with BC status as the outcome combining genetic effects and covariate model.	2a
Jantzen, 2021 [53]	Canada	Invasive BC	10, 18, 77 and 86 SNPs	Previously identified SNPs from 4 published studies [38,56,59,82].	Linear combinations of the risk-conferring variant alleles weighted by their effect sizes.	Available factors from existing models (BCRAT and IBIS).	Use of the iCARE package to sum the PRS and BCRAT score (relative hazard regression score). Use of the IBIS tool to incorporate shifted PRS scores.	4
Jia, 2020 [75]	UK	Overall BC	282 SNPs	Available SNPs in dataset from the 313 SNPs previously identified by Mavaddat et al. [12].	Sum of the product of the weight and the number of risk alleles for each risk variant across all selected risk variants per individual.	Not specified—Only family history of cancer in first-degree relatives was included.	Logistical models adjusted for genotype array types.	4
Lakeman, 2020 [24]	The Netherlands	Overall BC (In situ or invasive BC)	313 SNPs	Previously identified SNPS from Mavaddat et al. [12].	Cumulative effect of per- allele log OR (obtained from the BCAC) for BC associated with each SNP and multiplied by the number of risk alleles.	Available factors from an existing model (BOADICEA).	Use of BOADICEA version 5.	4
Lee, 2015 [54]	Singapore	Overall BC (In situ or invasive BC)	75 SNPs	SNPs identified in GWAS or in BCAC from the Asian populations assuming no interaction between SNPs, mammographic density and other risk factors.	Cumulative effect of log OR for each SNP multiplied by the number of risk alleles.	From an existing model (Gail) + predictors relevant to their population.	Cox proportional hazards model.	1a
Li, 2021 [73]	Australia	Invasive BC	313 SNPs	Previously identified SNPs based on GWAS published by the BCAC [10,12].	BOADICEA: Sum of the per allele log-OR multiplied by the allele counts for each SNP across variants and then normalized using population-based risk and allele frequency. IBIS: Multiplying the SNP-specific relative risk by the genotype-specific relative risk of BC, which estimates the average population relative risk accounting for the population-based risk and the allele frequency for the women’s genotype.	From existing models (BOADICEA v5.0.0 and IBIS V8b).	Use of BOADICEA version 5.0.0 and the Tyler-Cuzick model v.8b.	4
Maas, 2016 [55]	Various (Australia, Europe, USA)	Invasive BC	92 SNPs	SNPs identified in the BPC3 study and previously published SNPs.	Combination of a PRS24 assuming additive associations on the log scale after adjustments and a simulated PRS68 conditional on case-control status and family history, using the model estimates of the log-OR and the allele frequencies for the SNPs with an estimate of the log-OR for family history.	Established BC risk factors.	Multivariate logistic regression.	1a
Mealiffe, 2010 [57]	USA	Invasive BC, ER-positive, ER-negative	7 SNPs	Statistically significant SNPs in GWAS with correction for multiple testing and confirmed in an independent set of case controls.	Product of genotype relative risk value for each SNP based on a log-additive model.	Available factors from an existing model (BCRAT).	Multiplying 5-year Gail absolute risk estimates by SNP risk score.	1b
Olsen, 2021 [71]	Estonia	Overall BC	973 SNPs	Previously identified SNPs from Läll et al. [83].	Weighted average of the two strongest associated PRS (named metaGRS_2_), each PRS obtained by a linear combination of SNPs effect weighted by their log beta-coefficients.	Statistically significant predictors from a fully adjusted Cox model.	Cox proportional hazards model adjusted for age.	2a
Pal Choudhury, 2020 [58]	UK and USA	Overall BC	313 SNPs	Previously identified SNPS from Mavaddat et al. [12].	Cumulative effect for the total number of SNPS per allele OR associated with SNPs multiplied by allele dosage for SNPs.	From existing models (iCARE-Lit, iCARE-BPC3, BCRAT, IBIS).	Use of the iCARE, BCRAT and IBIS v8 models.	4
Pal Choudhury, 2021 [25]	UK	Overall BC	313 SNPs	Previously identified SNPS from Mavaddat et al. [12].	Cumulative effect for the total number of SNPS per allele OR associated with SNPs multiplied by allele dosage for SNPs.	From existing models (BOADICEA and Tyrer–Cuzick).	Use of BOADICEA version 5 as described by Lee et al. [13] and the IBIS tool (v.8) as described by Brentnall et al. [45]	4
Shieh, 2016 [59]	USA	Invasive BC	76 (Asian) and 83 SNPs	GWAS significant SNPs (*p* < 5 × 10^−8^) associated with invasive BC in White, Asian or Hispanic women.	Bayesian approach of the composite likelihood ratio representing the individual effects of each SNP assuming independence and no interaction between them.	From an existing model (fitted-BCSC).	Use version 2.0 of the BCSC model for multivariable regression analysis.	2a
Shieh, 2017 [60]	USA	ER-positive	83 SNPs	GWAS significant SNPs (*p* < 5 × 10^−8^) associated with invasive BC in White, Asian or Hispanic women.	Bayesian approach of the composite likelihood ratio representing the individual effects of each SNP assuming independence and no interaction between them.	From an existing model (BCSC v1).	Conditional logistic regression using a multivariable model.	2a
Starlard-Davenport, 2018 [61]	USA	Overall BC	75 SNPs	Previously identified SNPs from Mavaddat et al. [56]	Mealiffe et al. approach [57]. SNP-based relative risk score using ORs per-allele and risk allele frequency assuming independence of additive risks on the log-OR scale. Then, multiplying the adjusted risk values for each SNP.	From an existing model (BCRAT).	Multiplying the SNP-based score by the model’s predicted 5-year and lifetime absolute risk of BC.	4
Vachon, 2015 [63]	USA and Germany	Overall BC, invasive BC	76 SNPs	Previously identified SNPS from published studies.	Cumulative effect of the log OR for each SNP multiplied by the corresponding number of minor alleles.	From an existing model (BCSC).	Logistic regression.	1b
van Veen, 2018 [62]	UK	Overall BC (Invasive and ductal carcinoma in-situ)	18 SNPs	SNPs associated with BC in GWAS.	Multiplying the per-allele OR for each SNP and normalizing the risk by the average risk expected in the population using published minor allele frequencies.	From an existing model (Tyrer–Cuzick).	Multiplying Tyrer–Cuzick 10-year absolute risk by density residual and PRS assuming independence.	1a
Yang X., 2022 [23]	Sweden	Invasive BC	313 SNPs	Previously identified SNPs from Mavaddat et al. [12].	SNP was given a per-allele log OR in a log-additive model and derived and standardized by the mean and standard deviation.	From existing model (BOADICEA v.6)	Used BOADICEA V.6 with Swedish age-specific and calendar period-specific population incidences for invasive BC.	4
Yang Y., 2022 [72]	Various (China, Japan, Korea Shanghai)	Overall BC	111 and 263 SNPs	Race-specific SNPs from two published studies [12,84].	Three different approaches based on the cumulative effect of the allelic dosage multiplied by the corresponding weight of each SNP: (1) reported European PRS, (2) PRS based on SNPs identified by fine-mapping of GWAS-identified risk loci and (3) PRS-based on genome-wide risk prediction algorithms.	Established BC risk factors.	An integrated risk prediction model included the PRS and the non-genetic risk score (weighted value of each risk factor plus the weight of the interaction between BMI and menopause status) as independent predictors of BC (BC~*PRS* + *NgRS*).	3
Zheng, 2010 [65]	China	Overall BC	9 SNPs	Statistically significant SNPs associated with BC in GWAS.	Cumulative effect of the OR for each SNP multiplied by the number of SNPs replicated in the study.	Established BC risk factors.	Similar approach to Gail et al. [81] to estimate the absolute risk of C according to the risk factors that a woman carried [85].	1b

* Results for development not shown. Abbreviations: UK = United Kingdom; USA = United States of America; LR = likelihood ratio; OR = Odds ratio; SNP = Single nucleotide polymorphism; BCAC = Breast Cancer Association Consortium; BPC3 = Breast and Prostate Cancer Cohort Consortium; BCSC = Breast Cancer Surveillance Consortium; BOADICEA = Breast and Ovarian Analysis of Disease Incidence and Carrier Estimation Algorithm; BCRAT = Breast Cancer Risk Assessment Tool (Gail Model); COGS = Collaborative Oncological Gene-Environment Study; IBIS = International Breast Intervention Study (Tyrer–Cuzick model); iCARE-Lit = Individualized Coherent Absolute Risk Estimator based on literature review; iCARE-BPC3 = Individualized Coherent Absolute Risk Estimator based on BPC3 analysis. TRIPOD levels: 1a = development only; 1b = development and validation using resampling; 2a = random split sample development and validation; 2b = non-random split sample development and validation; 3 = development and validation using separate data; 4 = validation only.

**Table 2:** Main predictors used in breast cancer (BC) prediction models including a Polygenic Risk Score (PRS).

Author, Year	Age	Age at Mena-Rche	Age at Meno- Pause	Age at First Live Birth	No of Live Births	Parity	Family History of BC	No of Relatives with BC	Breast Biopsy	No of Biopsies	Breast Density	Meno- Pausal Status	HRT Use	OC Use	History of BBD	BMI	Alcohol Use	Smoking Status	Race/ Ethnicity	Atypical Hyper-Plasia	Heig-Ht
Allman, 2015 [43] BCRAT	X	X		X				X ^1^											X ^Ⴕ^	NA	
Allman, 2015 [43] ^a^ IBIS	X	X		X			NA	X ^1^											X ^Ⴕ^		
Allman, 2021 [44]	X						X ^1^												X ^Ⴕ^		
Brentnall, 2021 [45]	X			X		X	X ^1^				X ^b^					X			X		
Darabi, 2012 [46]		X		X			X ^1^		NA		X ^b^				X	X					
Dite, 2013 [47]	X ^‡^	X		X				X ^1^		NA									X	NA	
Dite, 2016 [48] BOADICEA	X	X					X ^1^														
Dite, 2016 [48] BRCAPRO	X						X ^1^														
Dite, 2016 [48] BCRAT	X	X		X			X ^1^		NA											NA	
Dite, 2016 [48] IBIS	X	X		X																NA	
Eriksson, 2020 [66] ^c^	X						X ^1^				X	X	X			X	X	X			
Evans, 2022 [69] ^d^	X			X				X ^1,2,ႵႵ^	X		X										X
Hou, 2022 [70]	X	X			X		X					X				X					
Hurson, 2021 [51] <50 years old	X ^‡^	X		X		X	X							X	X	X	X				X
Hurson, 2021 [51] ≥50 years	X ^‡^	X	X	X		X	X						X ^e^	X	X	NA	X				X
Husing, 2012 [52]		X	X	X	X							X ^f^	X			X ^f^	X	X			
Jantzen, 2021 [53] BCRAT	X	X		X				X ^1^	X										X		
Jantzen, 2021 [53] ^g^ IBIS V.8.0.b	X	X	X	X	X		X				NA		X							X	X
Jia, 2020 [75]							X ^1^														
Lakeman, 2020 [24]	X	X	X	X	X								X	X		X	X				X
Lee, 2015 [54]	X	X		X				X ^1^	X		X					X			X		
Li, 2021 [73] ^h^ BOADICEA	X	X	X	X	X	X	X ^1,2^				NA	X	X	X		X	X				X
Li, 2021 [73] ^i^ IBIS	X	X		X	X		X ^1,2^		NA		NA	X	X		X	X				NA	X
Maas, 2016 [55]		X	X	X		X	X					X	X			X	X	X			X
Mealiffe, 2010 [57]	X	X		X				X ^1^		X									X	NA	
Olsen, 2021 [71]	X																				
Pal Choudhury, 2020 [58] iCARE-Lit	X ^‡^	X	X	X		X	X ^1^		X				X	X	X	X	X				
Pal Choudhury, 2020 [58] iCARE-BPC3	X ^‡^	X	X	X		X	X ^1^					X	X			X	X	X			
Pal Choudhury, 2020 [58] BCRAT	X ^‡^	X		X			X ^1^	X ^1^		X					X					X	
Pal Choudhury, 2020 [58] IBIS ^j^	X ^‡^	X	X	X		X	X ^1,2,3^	X ^1,2,3^				X	X			X				X	X
Pal Choudhury, 2021 [25] BOADICEA ^k^	X ^‡^	X	X	X		X	X ^1^						X	X		X	X				X
Pal Choudhury, 2021 [25] Tyrer–Cuzick	X ^‡^	X	X	X		X	X ^1^					X	X ^e^			X				X	X
Shieh, 2016 [59]	X						X ^1^		X		X					X			X ^Ⴕ^		
Shieh, 2017 [60] ^l^	X						X ^1^		X		X								X		
Starlard-Davenport, 2018 [61]	X	X		X				X ^1^		NA										NA	
Vachon, 2015 [63]	X										X					X					
van Veen, 2018 [62]	X			X		X	X ^1^				X					X			X		
Yang X., 2022 [23]	X	X	X	X		X		X ^1^			X	X	X	X		X	X				X
Yang Y., 2022 [72] ^f,m^		X		X			X								X	X					
Zheng, 2010 [65]^m^		X		X		X	X ^1^								X	X					

Abbreviations: BDD = Benign breast disease; BMI = Body mass index; HRT = Hormone therapy replacement; NA = Information not available in the dataset often noted as unknown; OC = Oral contraceptive. Notes: ^1^ = First-degree relative with cancer; ^2^ = Second-degree relative with cancer; ^3^ = Third-degree relatives with cancer. ^Ⴕ^ = Results are presented separately for different ethnicities. ^ႵႵ^ Age of breast cancer diagnosis in affected first- and second-degree relatives is also collected. ^‡^ = Results are presented stratified by age. ^a^ = Also includes number of first-degree relatives with ovarian cancer; ^b^ = Presented as percentage mammographic density; ^c^ = Also includes microcalcifications and masses; ^d^ = Also includes a custom gene panel (*ATM*, *BARD1*, *BRCA1*, *BRCA2*, *CDH1*, *CHEK2*, *NF1*, *PALB2*, *PTEN*, *RAD50*, *RAD51C*, *RAD51D* and *TP53*) and weight; ^e^ = Also includes HRT type; ^f^ = Used as an interaction between BMI and menopausal status at baseline; ^g^ = Also includes weight and history of ovarian cancer; ^h^ = Also includes year of birth, age at cancer diagnosis for family history and history of ovarian cancer; ^i^ = Also includes history of lobular carcinoma in situ, age at cancer diagnosis for family history and history of ovarian cancer; ^j^ = Also includes hyperplasia, lobular carcinoma in situ (LCIS), age at onset of breast cancer in a relative, bilateral breast cancer in a relative, ovarian cancer in a relative and male breast cancer; ^k^ = BOADICEA model also includes information on explicit family history of breast and other cancers, genetic factors such as pathogenic variants and unobserved genetic effects, breast tumor pathology and demographic factors (see Lee et al. 2019 [13]); ^l^ = Other factors includes sex hormone levels of estradiol; ^m^ = Also includess waist-to-hip ratio.

## Data Availability

The data can be shared up on request.

## Supplementary Materials

The following supporting information can be downloaded at: https://www.mdpi.com/article/10.3390/cancers15225380/s1, Table S1. Example of search strategy; Table S2. Description of risk models and participants; Table S3. Predictive performance of models in individual studies.
