# Supplementary material for: A Systematic Review and Critical Assessment of Breast Cancer Risk Prediction Tools Incorporating a Polygenic Risk Score for the General Population
Source: Cancers (Basel). 2023 Nov 12;15(22):5380. doi: 10.3390/cancers15225380 (PMC10670420; doi:10.3390/cancers15225380)
Supplement: Supplementary file 1 [file cancers-15-05380-s001.zip › cancers-2639788-supplementary.pdf]

Supplementary Materials

Table S1. Example of search strategy

**Database**	**#**	**Concept**	**Search strategy**
Pubmed/ MEDLINE	#1	Breast cancer	("Breast Neoplasms"[Mesh]) OR (breast* cancer) OR (breast* cancer[tiab]) OR (breast* tumor) OR (breast* tumor*[tiab]) OR (breast* neoplasm*) OR (breast* neoplasm*[tiab]) OR (breast* carcinoma*) OR (breast* carcinoma*[tiab])
	#2	Polygenic risk score (PRS)	("Multifactorial Inheritance"[Mesh]) OR ("Genetic Predisposition to Disease"[Mesh]) OR (polygenic OR polygenic score*[tiab]) OR (polygenic score*) OR (polygenic risk score*[tiab]) OR (polygenic risk score*) OR (polygenic score*[OT]) OR (polygenic risk score [OT]) OR (polygenic inheritance) OR (multigenic inheritance)
	#3	Cancer prediction	("Prognosis"[Mesh]) OR ("Early Detection of Cancer"[Mesh]) OR (Cancer prediction) OR (Cancer prediction[tiab]) OR (prediction model*) OR (prediction model*[OT]) OR (prediction model*[tiab]) OR (prediction tool*[OT]) OR (prediction tool*[tiab]) OR (prediction tool*) OR (mathematical model*) OR (cancer probability) OR (cancer likelihood) OR (cancer chance*)

Table S2. Description of risk models and participants

Author, year	Name of model	Prediction time	Number of cases	Number of controls	Ancestry*
**Models based only on genetic risk factors**					
Allman, 2015a [43]	NA	5-year risk	421	7118	African American
Allman, 2015b [43]	NA	5-year risk	147	3216	Hispanic
Dite, 2016 [48] – ER-positive	NA	5-year risk	261	856	European ancestry
Dite, 2016 [48] – ER-negative	NA	5-year risk	155	856	European ancestry
Du, 2021 [49] – Overall BC	NA	Lifetime risk[†]	9,241	10,193	African ancestry
Du, 2021 [49] – ER-positive	NA	Lifetime risk[†]	4,299	10,193	African ancestry
Du, 2021 [49] – ER-negative	NA	Lifetime risk[†]	2,636	10,193	African ancestry
Gao, 2022 [67] – Overall BC	NA	10-year and lifetime[‡] risk	9235	10,184	African ancestry
Gao, 2022 [67] – ER-positive	NA	10-year and lifetime[‡] risk	4295	10,184	African ancestry
Gao, 2022 [67] – ER-negative	NA	10-year and lifetime[‡] risk	2635	10,184	African ancestry
Ho, 2020a [50] – Overall BC	NA	10-year and lifetime[‡] risk	15,755	16,493	Asian (Chinese, Indian, Malay)
Ho, 2020a [50] – ER-positive	NA	10-year and lifetime[‡] risk	10,477	16,483	Asian (Chinese, Indian, Malay)
Ho, 2020a [50] – ER-negative	NA	10-year and lifetime[‡] risk	4,764	16,483	Asian (Chinese, Indian, Malay)
Ho, 2020b [50] – Overall BC	NA	10-year and lifetime[‡] risk	1507	1212	Asian American
Ho, 2020b [50] – ER-positive	NA	10-year and lifetime[‡] risk	1022	1212	Asian American
Ho, 2020b [50] – ER-negative	NA	10-year and lifetime[‡] risk	280	1212	Asian American
Ho, 2022a [68]	Validation set	10-year and lifetime[‡] risk	6392	6638	East Asian
Ho, 2022b [68]	Prospective test set	10-year and lifetime[‡] risk	1592	89,898	East Asian
Ho, 2022c [68]	Validation set	10-year and lifetime[‡] risk	585	1018	South Asian (Indian)
Hou, 2022 [70] – Overall BC	Validation set	5-year risk	427	374	Asian
Hou, 2022 [70] – ER-positive	Validation set	5-year risk	290	374	Asian
Hou, 2022 [70] – ER-negative	Validation set	5-year risk	124	374	Asian
Liu, 2021a [74]	NA	Lifetime risk[‡]	3960	29,634	European ancestry
Liu, 2021b [74]	NA	Lifetime risk[‡]	274	3,527	African ancestry
Liu, 2021c [74]	NA	Lifetime risk[‡]	147	2,049	Latinx ancestry
Mavaddat, 2015 [56]	NA	10-year risk	33,673	33,381	European ancestry
Mavaddat, 2019a [12] – Overall BC	Validation set	10-year risk	5,159	5,285	European ancestry
Mavaddat, 2019a [12] – ER-positive	Validation set	10-year risk	4,233	5,285	European ancestry
Mavaddat, 2019a [12] – ER-negative	Validation set	10-year risk	926	5,285	European ancestry
Mavaddat, 2019b [12] – Overall BC	Prospective test set	10-year risk	11,428	18,323	European ancestry

Mavaddat, 2019b [12] – ER-positive	Prospective test set	10-year risk	7,992	18,323	European ancestry
Mavaddat, 2019b [12] – ER-negative	Prospective test set	10-year risk	1,259	18,323	European ancestry
Wen, 2016 [64]	NA	Lifetime risk[‡]	11,905	11,662	East Asian
Yang X., 2022 [23]	NA	5-year risk	676	15,502	European ancestry
Yang Y., 2022a [72]	Validation set	10-year risk	1426	1323	Asian
Yang Y., 2022b [72]	Prospective set	10-year risk	368	736	Asian
**Models based on genetic risk factors and non-genetic risk factors**					
Allman, 2015c [43]	BCRAT	5-year risk	421	7118	African American
Allman, 2015d [43]	IBIS	5-year risk	421	7118	African American
Allman, 2015e [43]	BCRAT	5-year risk	147	3216	Hispanic
Allman, 2015f [43]	IBIS	5-year risk	147	3216	Hispanic
Allman, 2021a [44]	Streamlined Gail model	5-year risk	416	7005	African American
Allman, 2021b [44]	Streamlined Gail model	5-year risk	750	405	European ancestry
Allman, 2021c [44]	Streamlined Gail model	5-year risk	147	3,210	Hispanic
Brentnall, 2020 [45] – Overall BC	Tyrer-Cuzick v6	10-year risk	405	1,668	European ancestry
Brentnall, 2020 [45] – ER-positive	Tyrer-Cuzick v6	10-year risk	353	1,668	European ancestry
Brentnall, 2020 [45] – ER-negative	Tyrer-Cuzick v6	10-year risk	39	1,668	European ancestry
Darabi, 2012 [46]	Gail model	5-year risk	1017	856	European ancestry
Dite, 2013** [47] – Overall BC	BCRAT	5-year risk	962[a]	463[b]	European ancestry
Dite, 2013 [47] – ER-positive	BCRAT	5-year risk	298	463	European ancestry
Dite, 2013 [47] – ER-negative	BCRAT	5-year risk	151	463	European ancestry
Dite, 2016a [48]	BOADICEA	5-year risk	750	405	European ancestry
Dite, 2016b [48]	BRCAPRO	5-year risk	750	405	European ancestry
Dite, 2016c [48]	BCRAT	5-year risk	750	405	European ancestry
Dite, 2016d [48]	IBIS	5-year risk	750	405	European ancestry
Eriksson, 2020 [66]	NA	2-year risk	974	9,376	European ancestry
Evans, 2022 [69]	Tyrer-Cuzick v8	10-year risk	340	1410	European ancestry
Hou, 2022b [70] – Overall BC	NA	5-year risk	431	376	Asian
Hou, 2022b [70] – ER-positive	NA	5-year risk	290	374	Asian
Hou, 2022b [70] – ER-negative	NA	5-year risk	124	374	Asian
Hou, 2022c [70] – Overall BC	Gail-2	5-year risk	431	376	Asian
Hou, 2022c [70] – ER-positive	Gail-2	5-year risk	290	374	Asian
Hou, 2022c [70] – ER-negative	Gail-2	5-year risk	124	374	Asian
Hurson, 2021** [51]	iCARE-Lit	5-year risk	6,811[c]	286,801[d]	European ancestry
Husing, 2012 [52] – Overall BC	BCRAT	5-year risk	6,009	7,827	European ancestry
Husing, 2012 [52] – ER-positive	BCRAT	5-year risk	3,920	7,827	European ancestry
Husing, 2012 [52] – ER-negative	BCRAT	5-year risk	1,059	7,827	European ancestry
Jantzen, 2021a [53]	BCRAT	5-year risk	131	4,424	European ancestry
Jantzen, 2021b [53]	IBIS V.8.0b	5-year risk	131	4,424	European ancestry
Jia, 2020 [75]	NA	5-year risk	4,340	210,096	European ancestry
Lakeman, 2020 [24]	BOADICEA v5	10-year risk	320	6,202	European ancestry
Lee, 2015 [54]	Gail model	10-year risk	680	23,481	Asian (Chinese, Indian, Malay, Others)
Li, 2021a** [73]	BOADICEA	5-year and 10-year risk	408[e]	3,098[f]	European ancestry
Li, 2021b** [73]	IBIS	5-year and 10-year risk	408[e]	3,098[f]	European ancestry

Maas, 2016 [55]	NA	Lifetime risk[‡]	17,171	19,862	European ancestry
Mealiffe, 2010 [57]	Gail model	5-year risk	1,664	1,636	European ancestry
Olsen, 2021 [71]	NA	3 and 5-year risk	185	30,127	European ancestry
Pal Choudhury, 2020a** [58]	iCARE-Lit	5-year risk	863[g]	63,148[h]	European ancestry
Pal Choudhury, 2020b** [58]	iCARE-BPC3	5-year risk	863[g]	63,148[h]	European ancestry
Pal Choudhury, 2020c** [58]	BCRAT	5-year risk	863[g]	63,148[h]	European ancestry
Pal Choudhury, 2020d** [58]	IBIS	5-year risk	863[g]	63,148[h]	European ancestry
Pal Choudhury, 2021a** [25]	BOADICEA v.5	5-year risk	619[i]	718[j]	European ancestry
Pal Choudhury, 2021b** [25]	Tyrer-Cuzick v.8	5-year risk	619[i]	718[j]	European ancestry
Shieh, 2016a [59]	BCSC v2	5-year risk	448	448	European ancestry, East Asian and Hispanic
Shieh, 2016b [59]	BCSC v2	5-year risk	387	387	European ancestry
Shieh, 2016c [59]	BCSC v2	5-year risk	51	51	East Asian
Shieh, 2017 [60]	BCSC	5-year risk	110	214	European ancestry
Starlard-Davenport, 2018 [61]	BCRAT	5-year risk and lifetime risk[++]	319	559	African American
Vachon, 2015 [63]	BCSC	5-year risk	456	1166	European ancestry
van Veen, 2018 [62]	Tyrer-Cuzick v6	10-year risk	466	8897	European ancestry
Yang X., 2022 [23]	BOADICEA V.6	5-year risk	280	5693	European ancestry
Yang Y., 2022b [72]	Prospective set	10-year risk	368	736	Asian
Zheng, 2010 [65]	NA	10-year risk	3039	3082	Asian

Abbreviations: BCSC = Breast Cancer Surveillance Consortium; BPC3 = Breast and Prostate Cancer Cohort Consortium; BOADICEA = Breast and Ovarian Analysis of Disease Incidence and Carrier Estimation Algorithm; BCRAT = Breast Cancer Risk Assessment Tool (Gail Model); IBIS = International Breast Intervention Study (Tyrer-Cuzick model); iCARE-Lit = Individualized Coherent Absolute Risk Estimator based on literature review; iCARE-BPC3 = Individualized Coherent Absolute Risk Estimator based on BPC3 analysis

Notes: A streamlined model includes first degree family history and age; NA indicates that the model was not based on a pre-existing tool

[*] Indicates the most prevalent ancestry group on which the PRS was derived;

[**] Models are presented stratified by age groups.

[+] Lifetime risk is an estimated risk until the age of 85; [++] Lifetime risk is an estimated risk until the age of 90;

[‡] Lifetime risk is an estimated risk until the age of 80;

[a] = 333, 332 and 307 cases were aged 35 to 39, 40 to 49 and 50 to 59, respectively; [b] = 182, 151 and 130 non cases were aged 35 to 39, 40 to 49 and 50 to 59, respectively; [c] = 891 cases were aged <50 and 5,920 were aged ≥50; [d] = 54,058 non cases were aged <50 and 232,743 were aged ≥50; [e] = 235 cases were aged <65 and 173 were age ≥65; [f] = 1,732 controls were aged <65 and 1,366 were age ≥65; [g] = 263 cases were aged <50 and 598 were aged ≥50; [h] = 27,967 non cases were aged <50 and 36,044 were aged ≥50; [i] = 207 cases were aged <50 and 412 were aged ≥50; [j] = 233 non cases were aged <50 and 485 were aged ≥50.

Table S3. Predictive performance of models in individual studies

Author, year	Measure of discrimination	Discriminative ability (95% CI)	Calibration	Reclassification (95% CI)
**Models based on genetic risk factors alone**				
Allman, 2015a [43]	AUC	0.550 (0.530 – 0.580)	x^2 = 6.0, P = 0.6	
Allman, 2015b [43]	AUC	0.590 (0.540 – 0.640)	x^2 = 20.8, P = 0.01	
Allman, 2021a [44]	AUC	0.550 (0.525 – 0.584)	NA	
Allman, 2021b [44]	AUC	0.612 (0.597 – 0.646)	NA	
Allman, 2021c [44]	AUC	0.590 (0.543 – 0.636)	NA	
Darabi, 2012 [46]	AUC	0.589 (0.563 – 0.614)	NA	
Dite, 2013 [47] – All ages	AUC	0.580 (0.540 – 0.610)	x^2 = 7.27, P = 0.5	
Dite, 2013 [47] – 35 to 39	AUC	0.600 (0.550 – 0.650)	x^2 = 12.89, P = 0.1	
Dite, 2013 [47] – 40 to 49	AUC	0.580 (0.530 – 0.640)	x^2 = 10.31, P = 0.2	
Dite, 2013 [47] – 50 to 59	AUC	0.540 (0.480 – 0.600)	x^2 = 3.80, P = 0.9	
Dite, 2016 [48] – Overall BC	AUC	0.610 (0.580 – 0.650)	x^2 = 11.4, P = 0.2	
Dite, 2016 [48] – ER-positive	AUC	0.650 (0.610 – 0.690)	NA	
Dite, 2016 [48] – ER-negative	AUC	0.560 (0.500 – 0.620)	NA	
Du, 2021a [49] – $PRS_{179\ SNPs}$	AUC	0.568 (0.560 – 0.576)	NA	
Du, 2021a [49] – $PRS_{313\ SNPs}$	AUC	0.571 (0.562 – 0.579)	NA	
Du, 2021b [49] – $PRS_{179\ SNPs}$	AUC	0.576 (0.566 – 0.585)	NA	
Du, 2021b [49] – $PRS_{313\ SNPs}$	AUC	0.588 (0.577 – 0.599)	NA	
Du, 2021c [49] – $PRS_{179\ SNPs}$	AUC	0.578 (0.564 – 0.591)	NA	
Du, 2021c [49] – $PRS_{313\ SNPs}$	AUC	0.562 (0.551 – 0.573)	NA	
Eriksson, 2020 [66]	AUC	0.640 (0.620 – 0.660)	NA	
Gao, 2022 [67] – Overall BC	AUC	0.581 (0.566 – 0.597)	NA	
Gao, 2022 [67] – ER-positive	AUC	0.608 (0.588 – 0.627)	NA	
Gao, 2022 [67] – ER-negative	AUC	0.576 (0.553 – 0.598)	NA	
Ho, 2020a [50] – Overall BC	AUC	0.613 (NA)	NA	
Ho, 2020a [50] – Overall BC (Chinese)	AUC	0.620 (0.600 – 0.630)	NA	
Ho, 2020a [50] – Overall BC (Indian)	AUC	0.600 (0.580 – 0.600)	NA	
Ho, 2020a [50] – Overall BC (Malay)	AUC	0.610 (0.590 – 0.640)	NA	
Ho, 2020a [50] – ER-positive	AUC	0.627 (NA)	NA	
Ho, 2020a [50] – ER-positive (Chinese)	AUC	0.630 (0.610 – 0.640)	NA	
Ho, 2020a [50] – ER-positive (Indian)	AUC	0.620 (0.600 – 0.650)	NA	
Ho, 2020a [50] – ER-positive (Malay)	AUC	0.630 (0.600 – 0.670)	NA	
Ho, 2020a [50] – ER-negative	AUC	0.594 (NA)	NA	
Ho, 2020a [50] – ER-negative (Chinese)	AUC	0.600 (0.580 – 0.610)	NA	
Ho, 2020a [50] – ER-negative (Indian)	AUC	0.570 (0.530 – 0.600)	NA	
Ho, 2020a [50] – ER-negative (Malay)	AUC	0.590 (0.540 – 0.630)	NA	
Ho, 2020b [50] – Overall BC	AUC	0.577 (NA)	NA	
Ho, 2020b [50] – ER-positive	AUC	0.586 (NA)	NA	
Ho, 2020b [50] – ER-negative	AUC	0.587 (NA)	NA	
Ho, 2022a [68] – PRS-CSx	AUC	0.636 (NA)	NA	
Ho, 2022b [68] – PRS-CSx	AUC	0.635 (NA)	NA	

Ho, 2022c [68] – PRS-CSx	AUC	0.633 (NA)	NA
Hou, 2022 [70] – Overall BC	AUC	0.601 (0.562 – 0.640)	E/O = 1.09 (0.77 – 1.41)
Hou, 2022 [70] – ER-positive	AUC	0.620 (0.577 – 0.663)	E/O = 1.09 (0.80 – 1.38)
Hou, 2022 [70] – ER-negative	AUC	0.555 (0.496 – 0.614)	E/O = 1.23 (0.18 – 2.28)
Hurson, 2021 [51] – <50	AUC	0.631 (0.610 – 0.651)	x^2 = 10.4, *P* = 0.32
Hurson, 2021 [51] – ≥50	AUC	0.622 (0.614 – 0.630)	x^2 = 11.0, *P* = 0.27
Husing, 2012 [52] – $PRS_{7\ SNPs}$	AUROC	0.564 (0.547 – 0.581)	NA
Husing, 2012 [52] – $PRS_{9\ SNPs}$	AUROC	0.569 (0.552 – 0.586)	NA
Husing, 2012 [52] – Overall BC $PRS_{18\ SNPs}$	AUROC	0.584 (0.567 – 0.600)	NA
Husing, 2012 [52] – ER-positive $PRS_{18\ SNPs}$	AUROC	0.595 (0.574 – 0.617)	NA
Husing, 2012 [52] – ER-negative $PRS_{18\ SNPs}$	AUROC	0.530 (0.492 – 0.567)	NA
Husing, 2012 [52] – Overall BC $PRS_{32\ SNPs}$	AUROC	0.583 (0.567 – 0.600)	NA
Husing, 2012 [52] – ER-positive $PRS_{32\ SNPs}$	AUROC	0.596 (0.574 – 0.618)	NA
Husing, 2012 [52] – ER-negative $PRS_{32\ SNPs}$	AUROC	0.530 (0.493 – 0.568)	NA
Jantzen, 2021 [53] – $PRS_{10\ SNPs}$	c-index	0.643 (0.581 – 0.704)	E/O = 0.81 (0.62 – 1.04) *P = 0.2270;* Slope = 1.1 (0.5 – 1.7)
Jantzen, 2021 [53] – $PRS_{18\ SNPs}$	c-index	0.634 (0.567 – 0.702)	E/O = 0.82 (0.63 – 1.06) *P = 0.1992;* Slope= 1.2 (0.6 – 1.8)
Jantzen, 2021 [53] – $PRS_{77\ SNPs}$	c-index	0.608 (0.530 – 0.685)	E/O = 0.83 (0.65 – 1.08) *P = 0.0984;* Slope = 0.9 (0.4 – 1.4)
Jantzen, 2021 [53] – $PRS_{86\ SNPs}$	c-index	0.626 (0.545 – 0.706)	E/O = 0.81 (0.63 – 1.05) *P = 0.1009;* Slope = 0.7 (0.4 – 1.8)
Jia, 2020 [75]	AUC	0.628 (0.620 – 0.637)	NA
Lakeman, 2020 [24] – <60	c-statistic	0.632 (0.580 – 0.690)	NA
Lakeman, 2020 [24] – 60 to 70	c-statistic	0.673 (0.610 – 0.730)	NA
Lakeman, 2020 [24] – ≥70	c-statistic	0.562 (0.480 – 0.620)	NA
Liu, 2021a [74] – $PRS_{313\ SNPs}$	AUC	0.590 (0.580 – 0.600)	NA
Liu, 2021a [74] – $PRS_{3,820\ SNPs}$	AUC	0.600 (0.590 – 0.610)	NA
Liu, 2021a [74] – $PRS_{5,218\ SNPs}$	AUC	0.610 (0.600 – 0.620)	NA
Liu, 2021b [74] – $PRS_{34\ SNPs}$	AUC	0.520 (0.480 – 0.550)	NA
Liu, 2021b [74] – $PRS_{75\ SNPs}$	AUC	0.500 (0.470 – 0.540)	NA
Liu, 2021c [74] – $PRS_{71\ SNPs}$	AUC	0.480 (0.430 – 0.530)	NA
Liu, 2021c [74] – $PRS_{180\ SNPs}$	AUC	0.540 (0.470 – 0.620)	NA
Maas, 2016 [55]	AUC	0.623 (NA)	NA
Mavaddat, 2015 [56]	c-statistic	0.622 (0.619 – 0.627)	NA
Mavaddat, 2019a [12] – Overall BC PRS_{77}	AUC	0.612 (NA)	NA
Mavaddat, 2019a [12] – Overall BC PRS_{313}	AUC	0.639 (NA)	NA
Mavaddat, 2019a [12] – Overall BC $PRS_{3,820}$	AUC	0.646 (NA)	NA
Mavaddat, 2019a [12] – ER-positive PRS_{77}	AUC	0.623 (NA)	NA

Mavaddat, 2019a [12] – ER-positive PRS_{313}	AUC	0.651(NA)	NA	
Mavaddat, 2019a [12] – ER-positive $PRS_{3,820}$	AUC	0.659 (NA)	NA	
Mavaddat, 2019a [12] – ER-negative PRS_{77}	AUC	0.596 (NA)	NA	
Mavaddat, 2019a [12] – ER-negative PRS_{313}	AUC	0.611 (NA)	NA	
Mavaddat, 2019a [12] – ER-negative $PRS_{3,820}$	AUC	0.611 (NA)	NA	
Mavaddat, 2019b [12] – Overall BC PRS_{77}	AUC	0.603 (NA)	NA	
Mavaddat, 2019b [12] – Overall BC PRS_{313}	AUC	0.630 (0.628–0.651)	*P < 0.05*	
Mavaddat, 2019b [12] – Overall BC $PRS_{3,820}$	AUC	0.636 (NA)	NA	
Mavaddat, 2019b [12] – ER-positive PRS_{77}	AUC	0.615 (NA)	NA	
Mavaddat, 2019b [12] – ER-positive PRS_{313}	AUC	0.641 (NA)	NA	
Mavaddat, 2019b [12] – ER-positive $PRS_{3,820}$	AUC	0.647 (NA)	NA	
Mavaddat, 2019b [12] – ER-negative PRS_{77}	AUC	0.584 (NA)	NA	
Mavaddat, 2019b [12] – ER-negative PRS_{313}	AUC	0.601 (NA)	NA	
Mavaddat, 2019b [12] – ER-negative $PRS_{3,820}$	AUC	0.600 (NA)	NA	
Mealiffe, 2010 [57] – Overall BC	AUC	0.587 (0.567 – 0.607)	*P* = 0.18	
Mealiffe, 2010 [57] – ER-positive	AUC	0.593 (0.572 – 0.614)	NA	
Mealiffe, 2010 [57] – ER-negative	AUC	0.541 (0.496 – 0.583)	NA	
Shieh, 2016a [59]	AUROC	0.600 (0.570 – 0.640)	x^2 = 975.7, *P = 0.42*	
Shieh, 2016b [59]	AUROC	0.590 (0.560 – 0.630)	NA	
Shieh, 2016c [59] – $PRS_{76\ SNPs}$	AUROC	0.640 (0.530 – 0.740)	NA	
Shieh, 2016c [59] – $PRS_{83\ SNPs}$	AUROC	0.620 (0.520 – 0.730)	NA	
Shieh, 2017 [60]	AUC	0.680 (0.610 – 0.750)	NA	
Starlard-Davenport, 2018 [61]	AUC	0.581 (0.541 – 0.620)	NA	
Vachon, 2015 [63]	AUC	0.676 (NA)	NA	
Wen, 2016 [64]	AUC	0.606 (NA)	NA	
Yang X., 2022 [23]	AUC	0.670 (0.640 – 0.690)	E/O = 1.06 (0.99 – 1.15) Slope = 1.02 (1.00 – 1.03)	
Yang Y., 2022a [72] – $PRS_{111\ SNPs}$	AUC	0.603 (0.582 – 0.624)	NA	
Yang Y., 2022a [72] – $PRS_{263\ SNPs}$	AUC	0.600 (0.579 – 0.621)	NA	
Yang Y., 2022b [72] – $PRS_{111\ SNPs}$	AUC	0.639 (0.604 – 0.674)	NA	
Yang Y., 2022b [72] – $PRS_{263\ SNPs}$	AUC	0.626 (0.592 – 0.661)	NA	
**Models based on genetic risk factors and clinical or other risk factors**				
Allman, 2015c [43]	AUC	0.590 (0.560 – 0.610)	x^2 = 9.9, *P* = 0.3	$NRI^{0.015,\ 0.02}$ = 0.033 (0.025 – 0.089)
Allman, 2015d [43]	AUC	0.550 (0.520 – 0.580)	x^2 = 6.9, *P* = 0.5	$NRI^{0.015,\ 0.02}$ = 0.060 (0.005 – 0.113)

Allman, 2015e [43]	AUC	0.610 (0.560 – 0.660)	x^2 = 4.7, *P* = 0.8	$NRI^{0.015, 0.02}$ = 0.082 (0.003 – 0.162)
Allman, 2015f [43]	AUC	0.590 (0.540 – 0.640)	x^2 = 3.9, *P* = 0.9	$NRI^{0.015, 0.02}$ = 0.181 (0.085 – 0.273)
Allman, 2021a [44]	AUC	0.570 (0.539 – 0.601)	NA	NA
Allman, 2021b [44]	AUC	0.639 (0.606 – 0.672)	NA	NA
Allman, 2021c [44]	AUC	0.601 (0.554 – 0.647)	NA	NA
Brentnall, 2020 [45] – Overall BC	aAUC	0.640 (0.610 – 0.670)	O/E = 1.06 (0.82 – 1.29)	NA
Brentnall, 2020 [45] – ER-positive	aAUC	0.650 (0.620 – 0.680)	O/E = 1.09 (0.84 – 1.34)	NA
Brentnall, 2020 [45] – ER-negative	aAUC	0.630 (0.540 – 0.710)	O/E = 0.87 (0.21 – 1.53)	NA
Darabi, 2012b [46]	AUC	0.619 (0.594 – 0.644)	x^2 = 22.82, *P* = 0.0036	$NRI_e^{0.02,0.04}$ = 0.170, *Z* = 5.750; *P* = 8.93 x 10^{-9}
Dite, 2013 [47] – Overall BC (All ages)	AUC	0.610 (0.580 – 0.640)	x^2 = 6.25, *P* = 0.6	$NRI^{0.015, 0.02}$ = 0.028, *P* = 0.5
Dite, 2013 [47] – Overall BC (35 to 39)	AUC	0.650 (0.600 – 0.700)	x^2 = 7.74, *P* = 0.5	$NRI^{0.015, 0.02}$ = 0.021, *P* = 0.4
Dite, 2013 [47] – Overall BC (40 to 49)	AUC	0.630 (0.570 – 0.690)	x^2 = 14.81, *P* = 0.06	$NRI^{0.015, 0.02}$ = 0.074, *P* = 0.4
Dite, 2013 [47] – Overall BC (50 to 59)	AUC	0.560 (0.510 – 0.620)	x^2 = 4.45, *P* = 0.8	$NRI^{0.015, 0.02}$ = -0.029, *P* = 0.5
Dite, 2013 [47] – ER-positive	AUC	0.610 (0.570 – 0.650)	NA	NA
Dite, 2013 [47] – ER-negative	AUC	0.550 (0.490 – 0.590)	NA	NA
Dite, 2016a [48]	AUC	0.700 (0.670 – 0.730)	x^2 = 9.9, *P* = 0.3	$NRI^{0.015, 0.02}$ = 0.040 (0.007 – 0.073)
Dite, 2016b [48]	AUC	0.690 (0.660 – 0.720)	x^2 = 8.9, *P* = 0.4	$NRI^{0.015, 0.02}$ = 0.063 (0.030 – 0.094)
Dite, 2016c [48]	AUC	0.670 (0.630 – 0.700)	x^2 = 7.0, *P* = 0.5	$NRI^{0.015, 0.02}$ = 0.066 (0.019 – 0.110)
Dite, 2016d [48]	AUC	0.630 (0.590 – 0.660)	x^2 = 7.2, *P* = 0.5	$NRI^{0.015, 0.02}$ = 0.052 (0.015 – 0.088)
Erickson, 2020 [66]	AUC	0.770 (0.750 – 0.790)	NA	NA
Evans, 2022 [69] – PRS_{18}	AUC	0.634 (0.602 – 0.667)	NA	NA
Evans, 2022 [69] – PRS_{143}	AUC	0.684 (0.652 – 0.715)	NA	NA
Evans, 2022 [69] – PRS_{313}	AUC	0.672 (0.641 – 0.704)	NA	NA
Hou, 2022b [70] – Overall BC	AUC	0.596 (0.557 – 0.635)	E/O = 1.17 (0.62 – 1.72)	NA
Hou, 2022b [70] – ER-positive	AUC	0.614 (0.571 – 0.657)	E/O = 1.12 (0.65 – 1.59)	NA
Hou, 2022b [70] – ER-negative	AUC	0.555 (0.496 – 0.614)	E/O = 1.15 (0.41 – 1.89)	NA
Hou, 2022c [70] – Overall BC	AUC	0.600 (0.561 – 0.639)	E/O = 1.13 (0.83-1.42)	NA
Hou, 2022c [70] – ER-positive	AUC	0.619 (0.576 – 0.662)	E/O = 1.11 (0.74-1.48)	NA
Hou, 2022c [70] – ER-negative	AUC	0.556 (0.497 – 0.615)	E/O = 1.11 (0.00-2.22)	NA
Hurson, 2021 [51] – <50	AUC	0.640 (0.620 – 0.660)	x^2 = 18.3, *P* = 0.03	$Reclassification^{0.03}$ (USA) = 12.3% of future cases
Hurson, 2021 [51] – ≥50	AUC	0.640 (0.632 – 0.647)	x^2 = 42.5, *P* < 0.01	$Reclassification^{0.03}$ (UK) = 15.7% of future cases
Husing, 2012 [52] – $PRS_{7\ SNPs}$	$AUROC_a$	0.591 (0.574 – 0.608)	NA	NA
Husing, 2012 [52] – $PRS_{9\ SNPs}$	$AUROC_a$	0.595 (0.579 – 0.612)	NA	NA
Husing, 2012 [52] – Overall BC $PRS_{18\ SNPs}$	$AUROC_a$	0.605 (0.589 – 0.622)	NA	NRI = 8.3%
Husing, 2012 [52] – ER-positive $PRS_{18\ SNPs}$	$AUROC_a$	0.618 (0.596 – 0.639)	NA	NA
Husing, 2012 [52] – ER-negative $PRS_{18\ SNPs}$	$AUROC_a$	0.554 (0.517 – 0.591)	NA	NA

Husing, 2012 [52] – Overall BC $PRS_{32\ SNPs}$	$AUROC_a$	0.604 (0.588 – 0.621)	NA	NA
Husing, 2012 [52] – ER-positive $PRS_{32\ SNPs}$	$AUROC_a$	0.618 (0.596 – 0.639)	NA	NA
Husing, 2012 [52] – ER-negative $PRS_{32\ SNPs}$	$AUROC_a$	0.554 (0.517 – 0.591)	NA	NA
Jantzen, 2021a [53] – $PRS_{10\ SNPs}$	C-index	0.636 (0.575 – 0.697)	E/O = 0.82 (0.64 – 1.06) *P = 0.048;* Slope = 0.7 (0.2 – 1.1)	NA
Jantzen, 2021a [53] – $PRS_{18\ SNPs}$	C-index	0.627 (0.559 – 0.695)	E/O = 0.83 (0.64 – 1.08) *P = 0.047;* Slope = 0.7 (0.3 – 1.1)	NA
Jantzen, 2021a [53] – $PRS_{77\ SNPs}$	C-index	0.614 (0.541 – 0.688)	E/O = 0.83 (0.64 – 1.07) *P = 0.161*; Slope = 0.6 (0.2 – 1.0)	NA
Jantzen, 2021a [53] – $PRS_{86\ SNPs}$	C-index	0.634 (0.559 – 0.695)	E/O = 0.86 (0.66 – 1.11) *P = 0.130*; Slope = 0.6 (0.2 – 1.0)	NA
Jantzen, 2021b [53] – $PRS_{10\ SNPs}$	C-index	0.642 (0.579 – 0.705)	E/O = 0.94 (0.73 – 1.22) *P = 0.627*; Slope = 0.9 (0.4 – 1.3)	NA
Jantzen, 2021b [53] – $PRS_{18\ SNPs}$	C-index	0.634 (0.564 – 0.703)	E/O = 0.94 (0.73 – 1.22) *P = 0.993*; Slope = 0.9 (0.4 – 1.4)	NA
Jantzen, 2021b [53] – $PRS_{77\ SNPs}$	C-index	0.627 (0.553 – 0.701)	E/O = 0.94 (0.73 – 1.22) *P = 0.470*; Slope = 0.8 (0.4 – 1.2)	NA
Jantzen, 2021b [53] – $PRS_{86\ SNPs}$	C-index	0.638 (0.564 – 0.703)	E/O = 0.95 (0.73 – 1.22) *P = 0.519*; Slope = 0.8 (0.4 – 1.2)	NA
Jia, 2020 [75]	AUC	0.633 (0.624 – 0.641)	NA	NA
Lakeman, 2020 [24]	c-statistic	0.653 (0.600 – 0.700)	Calibration plots	NA
Lee, 2015 [54]	AUC	0.680 (0.660 – 0.690)	NA	NA
Li, 2021a [73] – 5 year-risk	c-statistic	0.640 (0.610 – 0.670)	E/O = 0.87 (0.76 – 1.00)	NA
Li, 2021a [73] – <65	c-statistic	0.660 (0.620 – 0.690)	E/O = 0.97 (0.81 – 1.17)	NA
Li, 2021a [73] – ≥65	c-statistic	0.600 (0.560 – 0.650)	E/O = 0.75 (0.62 – 0.92)	NA
Li, 2021a [73] – 10-year risk	c-statistic	0.620 (0.590 – 0.640)	E/O = 0.85 (0.77 – 0.94)	NA
Li, 2021a [73] – <65	c-statistic	0.650 (0.620 – 0.680)	E/O = 0.94 (0.82 – 1.07)	NA
Li, 2021a [73] – ≥65	c-statistic	0.580 (0.530 – 0.620)	E/O = 0.73 (0.63 – 0.85)	NA
Li, 2021b [73] – 5 year-risk	c-statistic	0.630 (0.600 – 0.660)	E/O = 1.05 (0.91 – 1.20)	NA
Li, 2021b [73] – <65	c-statistic	0.640 (0.610 – 0.680)	E/O = 1.07 (0.89 – 1.30)	NA
Li, 2021b [73] – ≥65	c-statistic	0.620 (0.570 – 0.670)	E/O = 1.02 (0.83 – 1.25)	NA
Li, 2021b [73] – 10-year risk	c-statistic	0.620 (0.600 – 0.650)	E/O = 1.06 (0.95 – 1.17)	NA
Li, 2021b [73] – <65	c-statistic	0.640 (0.600 – 0.670)	E/O = 1.09 (0.95 – 1.25)	NA
Li, 2021b [73] – ≥65	c-statistic	0.600 (0.550 – 0.650)	E/O = 1.01 (0.86 – 1.18)	NA
Maas, 2016 [55]	AUC	0.648 (NA)	NA	NA
Mealiffe, 2010 [57] – Overall BC	AUC	0.594 (0.575 – 0.612)	*P* = 0.003	$NRI^{0.015,\ 0.02}$ = 0.085, *Z* = 4.3; *P* = 1.0 x 10^{-5}
Mealiffe, 2010 [57] – ER-positive	AUC	0.605 (0.583 – 0.625)	NA	NA
Mealiffe, 2010 [57] – ER-negative	AUC	0.521 (0.478 – 0.567)	NA	NA
Olsen, 2021 [71] – 3-year risk	AUC	0.720 (0.675 – 0.765)	Calibration plots	$NRI^{0.01}$ = 0.09 (0.02 – 0.16), *p* = 0.01

Olsen, 2021 [71] – 5-year risk	AUC	0.704 (0.670 – 0.737)	Calibration plots	$NRI^{0.01}$ = 0.05 (0.02 – 0.12), *p* = 0.17
Pal Choudhury, 2020a [58] – <50	AUC	0.654 (0.621 – 0.687)	E/O = 0.98 (0.87 – 1.11)	NA
Pal Choudhury, 2020a [58] – ≥50	AUC	0.622 (0.600 – 0.645)	E/O = 1.13 (1.04 – 1.22)	NA
Pal Choudhury, 2020b [58] – ≥50	AUC	0.602 (0.580 – 0.624)	E/O = 1.00 (0.93 – 1.09)	NA
Pal Choudhury, 2020c [58] – <50	AUC	0.640 (0.606 – 0.674)	E/O = 0.85 (0.75 – 0.95)	NA
Pal Choudhury, 2020c [58] – ≥50	AUC	0.582 (0.558 – 0.605)	E/O = 0.95 (0.88 – 1.03)	NA
Pal Choudhury, 2020d [58] – <50	AUC	0.646 (0.613 – 0.679)	E/O = 1.14 (1.01 – 1.29)	NA
Pal Choudhury, 2020d [58] – ≥50	AUC	0.614 (0.592 – 0.636)	E/O = 1.13 (1.05 – 1.23)	NA
Pal Choudhury, 2021a [25] – <50	AUC	0.697 (0.641 – 0.752)	E/O = 0.79 (0.64 – 0.98) x^2 = 17.8, *P* = 0.04 Slope = 0.92 (0.56 – 1.29)	NA
Pal Choudhury, 2021a [25] – ≥50	AUC	0.646 (0.609 – 0.682)	E/O = 0.88 (0.77 – 1.00) x^2 = 15.5, *P* = 0.08 Slope = 0.90 (0.57 – 1.24)	NA
Pal Choudhury, 2021b [25] – <50	AUC	0.694 (0.638 – 0.750)	E/O = 1.11 (0.90 – 1.38) x^2 = 23.3, *P* = 0.01 Slope = 0.58 (0.36 – 0.81)	NA
Pal Choudhury, 2021b [25] – ≥50	AUC	0.639 (0.602 – 0.676)	E/O = 1.18 (1.04 – 1.35) x^2 = 57.9, *P* < 0.01 Slope = 0.45 (0.16 – 0.73)	NA
Shieh, 2016a [59]	AUC	0.650 (0.610 – 0.680)	x^2 = 937.0, *P* = 0.41	NA
Shieh, 2016b [59]	AUC	0.630 (0.590 – 0.670)	NA	NA
Shieh, 2016c [59]	AUC	0.720 (0.620 – 0.820)	NA	NA
Shieh, 2017 [60]	AUC	0.720 (0.650 – 0.790)	NA	NA
Starlard-Davenport, 2018 [61] – 5-year risk	AUC	0.679 (0.642 – 0.716)	NA	$NRI^{0.017}$ = 0.020 (-0.040 – 0.080)
Starlard-Davenport, 2018 [61] – Lifetime risk	AUC	0.658 (0.619 – 0.696)	NA	$NRI^{0.2}$ = 0.034 (0.000 – 0.070)
Vachon, 2015 [63]	AUC	0.690 (0.640 – 0.730)	NA	$NRI_{e}^{0.03}$= 0.11 (0.07 – 0.15) $NRI_{ne}^{0.03}$ = 0.02 (-0.01 – 0.05)
van Veen, 2018 [62]	AUC	0.670 (0.620 – 0.710)	E/O = 0.98 (0.69 – 1.28)	NA
Yang X, 2022 [23]	AUC	0.700 (0.660 – 0.730)	E/O = 0.88 (0.75 – 1.04) Slope = 0.97 (0.95 – 0.99)	Reclassification table at 3% threshold
Yang Y., 2022b [72] – $PRS_{111\ SNPs}$	AUC	0.648 (0.613 – 0.682)	NA	NA
Yang Y., 2022b [72] – $PRS_{263\ SNPs}$	AUC	0.632 (0.597 – 0.666)	NA	NA
Zheng, 2010 [65]	c-statistic	0.630 (NA)	NA	Reclassification table at 1.5% threshold

Notes: x^2 = Chi-square goodness-of-fit-test statistics; *P* = goodness-of-fit p-value; *Z* = asymptomatic Z-test testing the null hypothesis that the net reclassification improvement is equal to 0.

Abbreviations: aAUC : Area under the adjusted receiver operating characteristic; AUC: Area under the receiver operating characteristic curve; $AUROC_a$: Covariate-adjusted area under the receiver operating characteristic curve; c-statistics : concordance statistics E/O = expected to observed ratio; NA: Information not available; NRI: Net reclassification improvement; NRI_e = Net proportion index for events; NRI_{ne} = Net proportion indexfor non-events; PRS-CSx : Extension of the Bayesian polygenic prediction method (https://github.com/getian107/PRScsx)
